# Transcriptome analysis reveals regulatory networks underlying differential susceptibility to *Botrytis cinerea* in response to nitrogen availability in *Solanum lycopersicum*

**DOI:** 10.3389/fpls.2015.00911

**Published:** 2015-11-04

**Authors:** Andrea Vega, Paulo Canessa, Gustavo Hoppe, Ignacio Retamal, Tomas C. Moyano, Javier Canales, Rodrigo A. Gutiérrez, Joselyn Rubilar

**Affiliations:** ^1^Facultad de Agronomía e Ingeniería Forestal, Pontificia Universidad Católica de ChileSantiago, Chile; ^2^Millennium Nucleus Center for Plant Systems and Synthetic BiologySantiago, Chile; ^3^Departamento de Genética Molecular y Microbiología, Facultad de Ciencias Biológicas, Pontificia Universidad Católica de ChileSantiago, Chile; ^4^Millennium Nucleus for Fungal Integrative and Synthetic BiologySantiago, Chile; ^5^Facultad de Ciencias, Instituto de Bioquímica y Microbiología, Universidad Austral de ChileValdivia, Chile

**Keywords:** nitrate nutrition, tomato, defense mechanisms, jasmonic acid, gene network analysis, ethylene signaling, microarray analysis

## Abstract

Nitrogen (N) is one of the main limiting nutrients for plant growth and crop yield. It is well documented that changes in nitrate availability, the main N source found in agricultural soils, influences a myriad of developmental programs and processes including the plant defense response. Indeed, many agronomical reports indicate that the plant N nutritional status influences their ability to respond effectively when challenged by different pathogens. However, the molecular mechanisms involved in N-modulation of plant susceptibility to pathogens are poorly characterized. In this work, we show that *Solanum lycopersicum* defense response to the necrotrophic fungus *Botrytis cinerea* is affected by plant N availability, with higher susceptibility in nitrate-limiting conditions. Global gene expression responses of tomato against *B. cinerea* under contrasting nitrate conditions reveals that plant primary metabolism is affected by the fungal infection regardless of N regimes. This result suggests that differential susceptibility to pathogen attack under contrasting N conditions is not only explained by a metabolic alteration. We used a systems biology approach to identify the transcriptional regulatory network implicated in plant response to the fungus infection under contrasting nitrate conditions. Interestingly, hub genes in this network are known key transcription factors involved in ethylene and jasmonic acid signaling. This result positions these hormones as key integrators of nitrate and defense against *B. cinerea* in tomato plants. Our results provide insights into potential crosstalk mechanisms between necrotrophic defense response and N status in plants.

## Introduction

Nitrogen (N) is an essential macronutrient whose availability significantly impacts plant growth and development. Since natural environments and agricultural fields have limited amounts of N, the production of high-yielding crops relies on the application of large quantities of nitrogenous fertilizers, which come at considerable economic (Good et al., 2004) and environmental costs (Hirel et al., 2011).

The relevance of N for plants is clearly exemplified by its effects on leaf growth (von Wirén et al., 2000), senescence (Vanacker et al., 2006), root system architecture (Zhang et al., 2007; Vidal et al., 2010) and flowering time (Castro Marín et al., 2010). Besides growth and developmental effects, it is also clear that N nutrition can impact the plant's ability to cope with environmental challenges such as plant pathogen attacks (Snoeijers et al., 2000; Walters and Bingham, 2007; Dordas, 2008; Fagard et al., 2014). Different studies have shown that N availability impacts the outcome of plant-pathogen interactions, although the mechanisms underlying this connection are poorly understood, and the effect of N on this process is highly dependent on the crop being studied and on the particular life style of the pathogen involved (Snoeijers et al., 2000; Walters and Bingham, 2007; Dordas, 2008; Fagard et al., 2014). Consequently, it is difficult to derive general rules for the role of N in this process.

The plant defense response is a complex biological process involving numerous changes at the biochemical, physiological, and molecular (transcriptional) level, all governed by an intricate grid of hierarchical and regulatory interactions (Windram et al., 2014). These defense mechanisms are triggered partly by the defense hormones salicylic acid (SA) and jasmonic acid (JA) (Thomma et al., 2001; Glazebrook, 2005; Pieterse et al., 2009; Caarls et al., 2015). In general terms, SA is involved in resistance against biotrophic pathogens (Vlot et al., 2009), while JA participates in the regulation of defense response against necrotrophic pathogens and insects (Farmer et al., 2003).

Since plant defense is an active and energetically costly response mechanism, it is expected that the metabolic state of the plant plays a fundamental role in the outcome of the plant-pathogen interaction. A few agronomic reports indicate that high N availability increases the incidence of crop diseases (Hoffland et al., 2000; Olesen et al., 2003; Ballini et al., 2013). On the other hand, some studies report that a reduction in N fertilization increases disease severity (Hoffland et al., 1999; Long et al., 2007; Linquist et al., 2008). In the model plant *Arabidopsis thaliana*, proteins involved in plant resistance to infections are up-regulated in response to changes in N levels (Lau and Hamer, 1996; Dietrich et al., 2004). In the case of *Botrytis cinerea*, one of the most important fungal plant pathogens with regard to both its scientific and agronomic importance (Dean et al., 2012), plant N status can either promote or impede infection, depending on the plant species. For instance, while high N fertilization rates increase disease severity in legumes (Davidson et al., 2007) and lettuce (Lecompte et al., 2013), elevated N concentrations result in reduced susceptibility to this fungus in tomato (Hoffland et al., 1999; Lecompte et al., 2010). As there appears to be a trade-off between plant growth and defense responses (Walters and Heil, 2007), an intricate interconnection between metabolic and stress signaling pathways is required for proper and efficient resource allocation (Hey et al., 2010). The relationship between N metabolism and plant defense responses however, has not been analyzed in detail, although it has recently been recognized that this interconnection may shed new light on the complexity of plant defense strategies (Fagard et al., 2014).

In this study, we analyzed changes in global gene expression patterns during *B. cinerea* infections in tomato plants grown under contrasting nitrate regimes, with the goal of characterizing the interaction between N supply and defense responses at molecular level. High nitrate availability reduced plant susceptibility to this fungus when measured in leaves and tomato fruits. Global gene expression patterns confirmed that the tomato plant defense response is affected by nitrate availability. To get a broader overview of the interconnection between N metabolism and the plant defense response, a gene network analysis was performed. This strategy identified a transcriptional regulatory network controlling plant susceptibility to this fungus depending on nitrate condition. After validating a network-derived prediction by RT-qPCR in leaves and fruits infected by the fungus, we conclude that the expression of key transcription factors (TFs) involved in ethylene (ET) and JA signaling is modulated by the plant N status when tomato is infected by *B. cinerea*, suggesting that these hormones play a role in the nitrate-defense response interaction in tomato. Our study identified crosstalk points between N-nutrition, defense response and the ET/JA pathways in plants.

## Results

### Plant nitrate regimes alter the progression of *B. cinerea* infection in tomato

As a first step to evaluate a connection between plant nitrate availability and plant response to pathogen infection, we evaluated the growth of *S. lycopercicum cv. MicroTom* under N (nitrate) conditions that ranged from limiting to sufficient. Tomato plants were grown in pots with vermiculite, an inert growing substrate without N sources, and irrigated with a complete mineral nutrient solution without N supplemented with 2, 4, 6, or 12 mM nitrate (final concentration). As expected, nitrate concentration had a significant impact on tomato plant growth. Maximal growth was attained with 6 mM (N-sufficient condition) under our experimental conditions, and 2 mM and 4 mM nitrate produced severe and mild growth phenotypes, respectively (N-limiting conditions). To evaluate higher N input, tomato plants were grown using 12 mM nitrate. The amount of shoot biomass was reduced upon lowering the amount of nitrate to that normally present in the N-sufficient condition (6 mM), while higher concentrations did not lead to significant changes (Figure S1).

**Figure 1:**
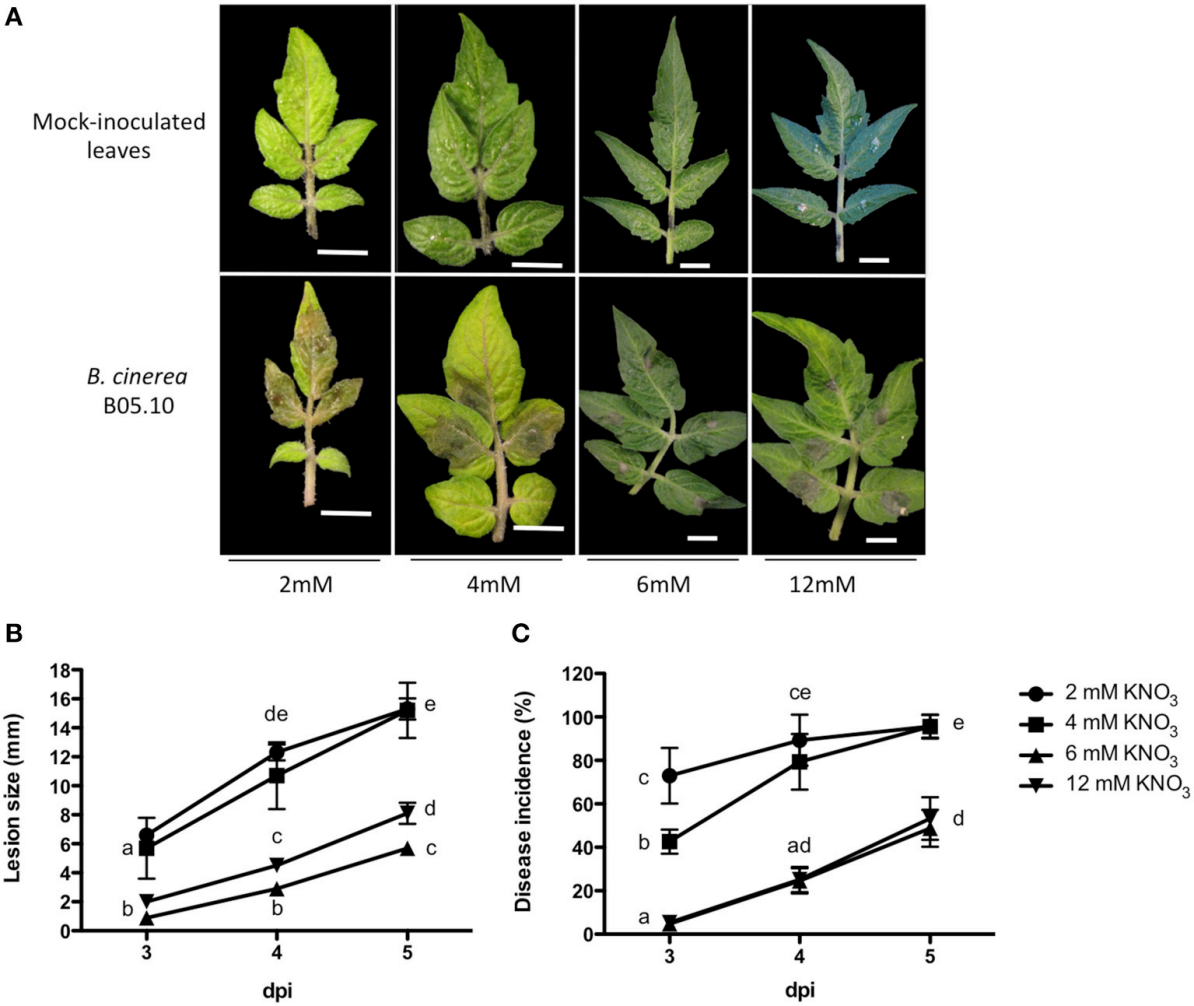
**Tomato leaves susceptibility to *B. cinerea* in plants grown under different nitrate concentrations**. **(A)** Representative inoculated leaves (3 dpi) for each nitrate condition used throughout this study. **(B)** Lesion size, measured as diameter of expanding lesions, for the indicated nitrate concentrations. **(C)** Disease incidence (percentage of leaf area with disease symptoms). Different letters indicate significant differences among treatments at a given time point (*p* ≤ 0.05; error bars indicate SEM; *n* = 6).

To evaluate whether contrasting nitrate concentrations impact the susceptibility of tomato plants to fungal infection, plants were grown under 2, 4, 6, or 12 mM nitrate as the only N source and challenged with *B. cinerea*. Leaves *in planta* were inoculated with an aqueous suspension of 5 × 10^3^ conidia. Typical *B. cinerea* symptoms, such as necrotic lesions, were observed in leaves under all N regimes. The first visual symptoms of the infections were detected 2 days post inoculation (dpi), at which time darkening of the leaf surface under the inoculum was observed (data not shown). As shown in Figure 1, disease symptoms developed faster in plants grown under N-limiting than in N-sufficient conditions. At 3 dpi, larger lesions were observed under N-limiting conditions, with evident tissue maceration surrounding primary infection sites in leaves (Figure 1A). Conversely, only discrete necrotic lesions were observed under N-sufficient conditions (Figure 1A). Even though, disease symptoms (expanding necrosis, chlorosis, and tissue maceration) were observed in leaves from plants grown under all N regimes at 5 dpi (data not shown), the size of the lesions and the percentage of the leaf exhibiting symptoms were always larger in leaves from plants grown under N-limiting conditions (Figures 1B,C).

To quantitatively assess fungal growth on plant tissue, we employed a quantitative PCR (qPCR) assay based on the relative quantification of fungal and plant DNA in infected plants, as described (Gachon and Saindrenan, 2004). As shown in Figure S2A, and consistent with the results shown in Figure 1, an increase in fungal growth was observed in all N-conditions as disease progressed, with larger quantities in infected plants grown under N-limiting conditions.

**Figure 2:**
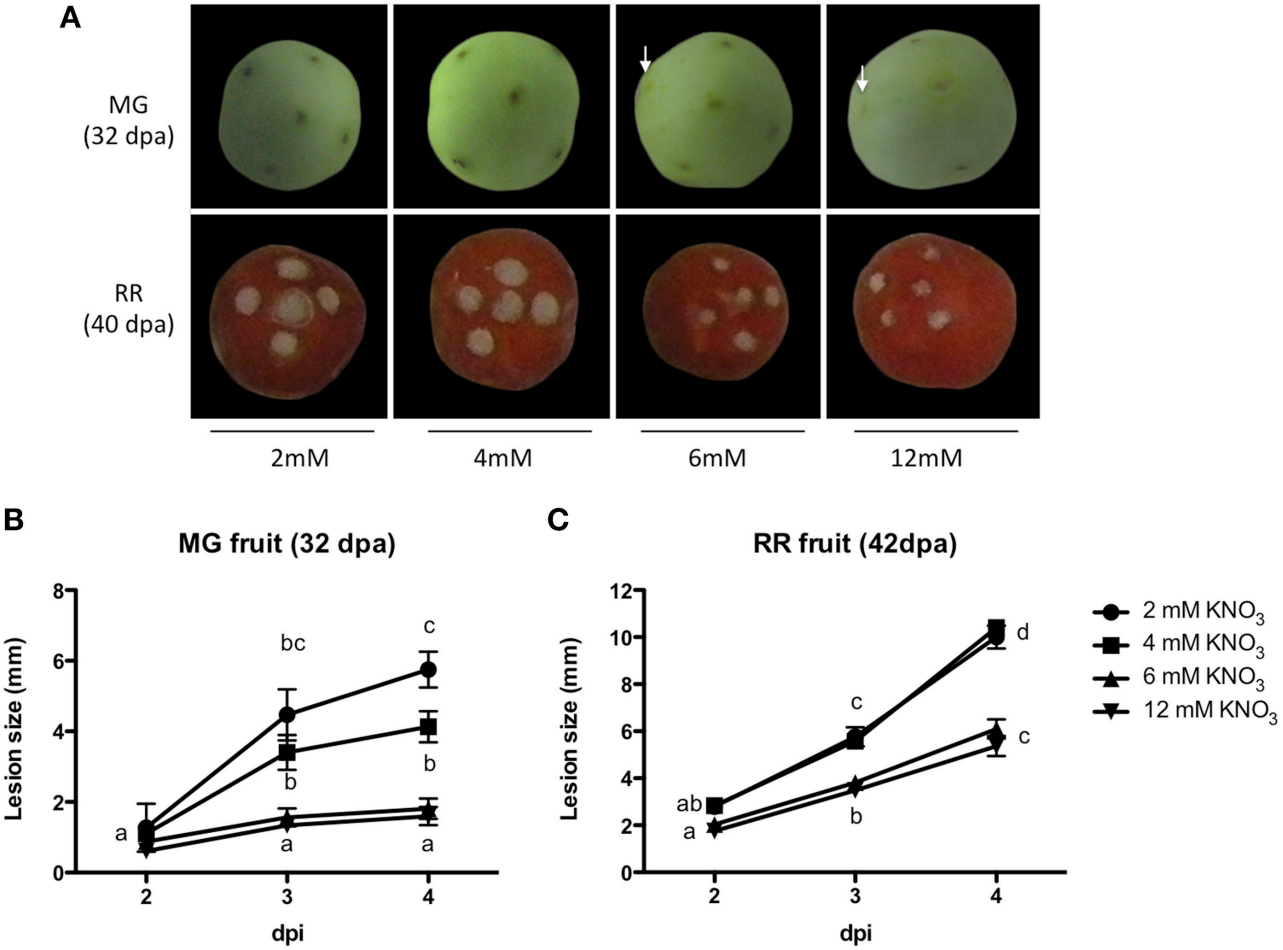
**Tomato fruits susceptibility to *B. cinerea* obtained from plants grown under different nitrate concentrations**. **(A)** Representative inoculated fruits (3 dpi) for each nitrate concentration and ripening stage (dpa, days post-anthesis). **(B,C)** Lesion size (diameter) for inoculated MG and RR fruits, respectively. For MG fruits, white arrows indicate the site of infection with minimal necrotic lesion. Different letters indicate significant differences among treatments at a given time point (*p* ≤ 0.05; error bars indicate SEM; *n* = 4).

Previous studies have shown that nitrate responses are organ and developmental stage-dependent (Wang et al., 2003, 2004; Vidal et al., 2014). To evaluate whether there was a difference in the aforementioned results when fruits instead of vegetative plant tissue was used, and considering that ripening promotes fruit susceptibility to pathogens (Alba et al., 2005; Giovannoni, 2007; Cantu et al., 2009), we evaluated the response of unripe green and ripe red tomato fruits to *B. cinerea* infection. Figure 2 shows disease symptoms and lesion progression in two developmental stages known as “mature green” (MG) and “red ripe” (RR) fruits obtained from plants grown under the same N conditions described above. Under all nitrate conditions evaluated, MG tomatoes were significantly less susceptible to *B. cinerea* compared to RR fruits. When MG tomato fruits were inoculated, a small necrotic lesion was observed at the site of infection 3 dpi (Figure 2A). On RR tomato fruits, on the other hand, tissue rotting, and fungal growth were already evident 3 dpi and extended into the pericarp tissue (Figure 2A). The severity of the disease symptoms (Figures 2B,C) and the accumulation of fungal biomass (Figures S2B,C) in infected MG and RR fruits from plants grown under N-limiting conditions were larger than those obtained from a N-sufficient regime. These results indicate that plants grown under N-limiting conditions exhibit enhanced susceptibility to *B. cinerea* infection.

### Global gene expression analysis supports a connection between plant N status and susceptibly to fungal infection in tomato

To better understand the molecular changes underlying the impact of nitrate availability on plant susceptibility to *B. cinerea*, we performed transcriptome-profiling assays on mock-treated and infected plants grown under severe limiting (2 mM) and sufficient (6 mM) N conditions, using GeneChip Tomato Genome Arrays (Affymetrix). We also included 12 mM nitrate in our analysis, as a higher N input. These experiments were performed using shoots since most plant defense responses have been described in this particular vegetative tissue (Glazebrook, 2005). As shown in Figure S3, our analysis suggests a significant interaction between the plant N-status and *B. cinerea* infection (Two-way ANOVA, *p* < 0.01), where 2110 genes showed significant Nitrogen/*B. cinerea* (N/B) interaction, representing approximately 4.7% of the analyzed tomato transcriptome (Table S1). Since this result suggests a nitrate and defense signaling crosstalk in response to *B. cinerea* infection, this set of genes was selected for further analysis.

**Figure 3:**
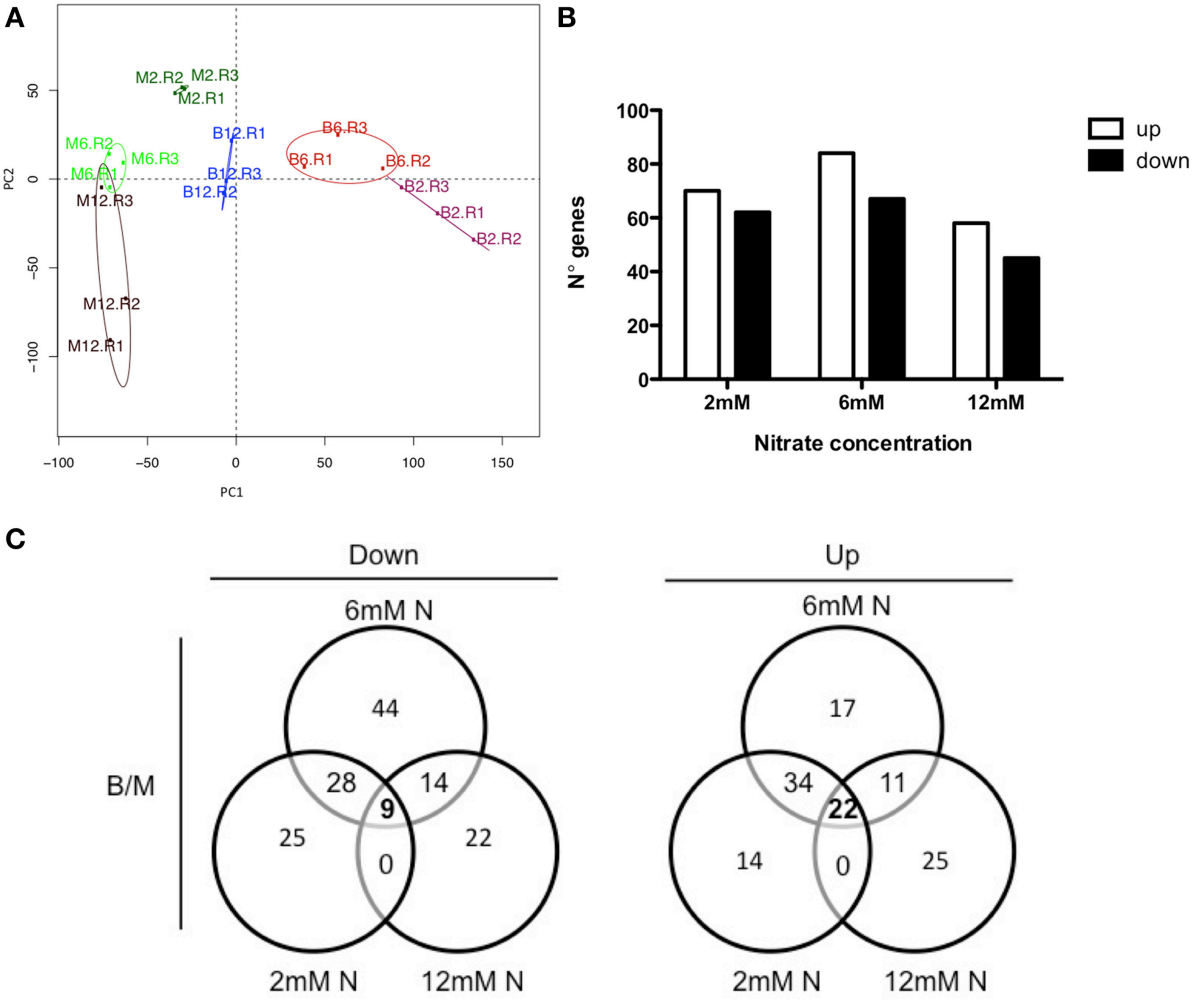
**The plant N-availability affects global gene expression in response to *B. cinerea* infection in tomato**. **(A)** Principal component analysis (PCA) of RMA-normalized global gene expression data demonstrate a connection between plant N-status and *B. cinerea* susceptibility. Colored dots denote each biological replicate. “M” and “B” indicate mock-treated and *B. cinerea*-infected plants, respectively, while the number next to each dot indicate the N concentration. **(B)** Number of genes that are either up- or down-regulated by the *B. cinerea* infection in each N condition analyzed in comparison to mock-inoculated plants (ANOVA; Rank-product, adjusted *p* < 0.05). **(C)** Venn diagrams of the data presented in **(B)** showing the overlap of genes that are either up- or down-regulated in tomato inoculated with *B. cinerea* in each N condition comparing with corresponding mock-treated samples (B/M).

Principal component analysis (PCA) (Figure 3A) showed that the first principal component (PC1) accounted for 45% of the total variation and segregated mock (M) from infected (B) samples, regardless of N-conditions. In addition, PC1 also allows the differentiation between N-status in *B. cinerea*-infected samples. The second principal component (PC2) accounted for 13% of the variation, and differentiated limiting vs. sufficient plant N-status in mock-treated samples. Indeed, mock-treated samples from plants grown under N-limiting conditions (2 mM nitrate) cluster more closely with *B. cinerea*-infected samples, rather than with non-infected samples, under both N-sufficient concentrations (6 mM and 12 mM nitrate). This result suggests that the transcriptome state of infected plants may be similar to N-limiting conditions. Moreover, the total number of differentially expressed genes (DEG) in response to pathogen infection was comparable among all N conditions evaluated (Figure 3B). For DEG analysis, genes with significant N/B model were used for a pairwise comparison between mock and infected samples in each N-condition, using Rank-products (*p* < 0.05). However, only 18% of up-regulated (22/123) genes were common to all N concentrations analyzed (Figure 3C). Although, the susceptibility phenotypes of plant grown under 2 mM and 6 mM nitrate were different in our experimental conditions, these N-conditions present more common up-regulated and down-regulated genes when compared with plants grown in 12 mM nitrate. These results suggest that higher N-input also affect plant defense response, indicating a complex link between N-metabolism and *B. cinerea* infections.

### Differentially expressed genes under N-sufficient condition supports better defense response in tomato

**Figure 4:**
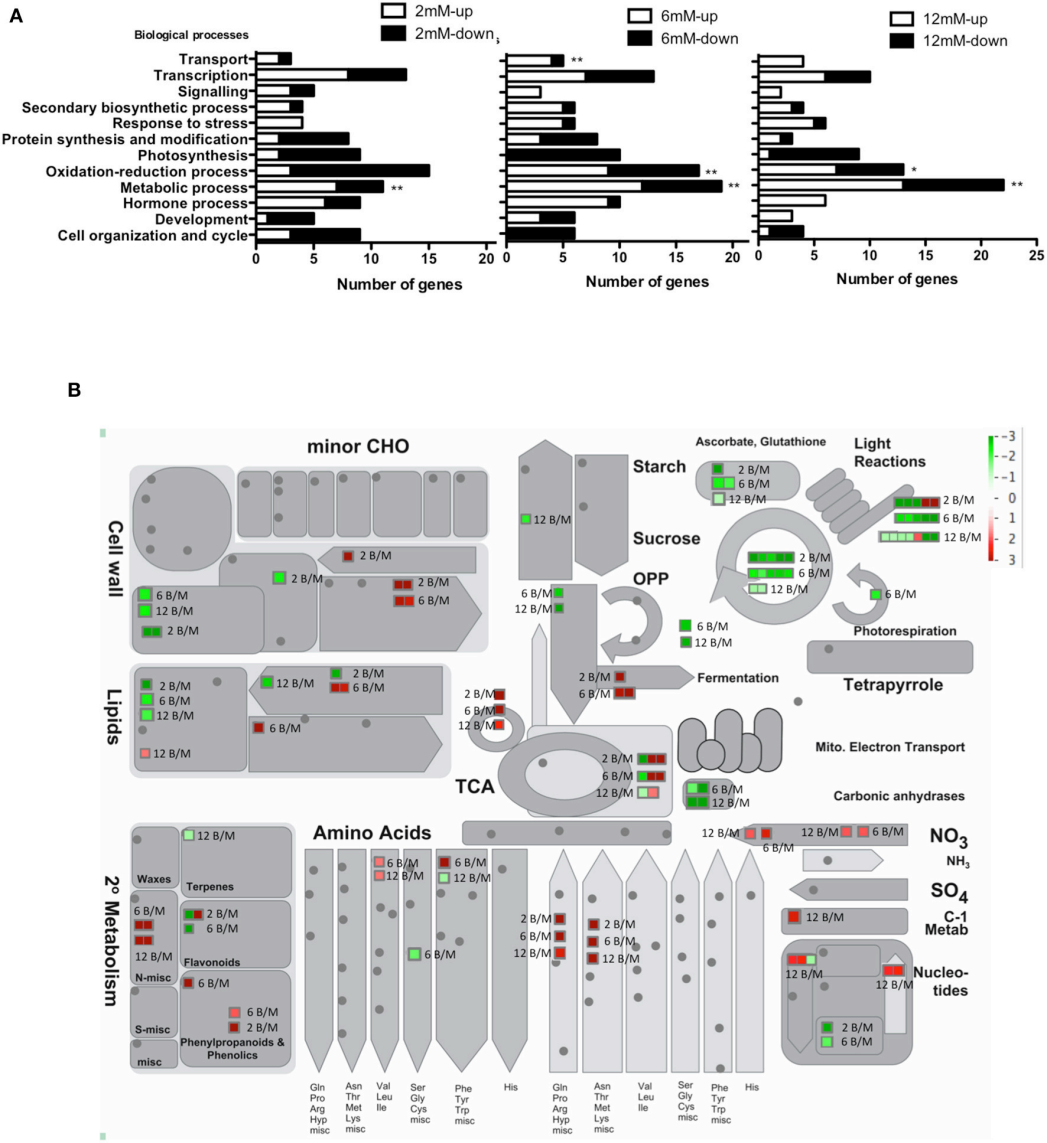
**Biological processes affected in response to *B. cinerea* infection under different N conditions**. **(A)** The distribution of genes into different GO categories for biological processes is shown. The number of genes induced or repressed (in white and black, respectively) by the fungal infection depending on the N condition used relative to mock-treated plants is presented. Only genes with significant differential expression (*p* < 0.05) were plotted, corresponding to 132, 151, and 103 genes (2, 6, and 12 mM nitrate, respectively). Overrepresented categories compared to the entire set on the Tomato genome array are indicated with asterisks (^**^p < 0.01, ^*^p < 0.05). **(B)** MapMan software was used to provide a snapshot of modulated genes over the main metabolic pathways. DEGs were binned to MapMan functional categories and Log2 fold changes values for each gene upon *B. cinerea* infection referred to mock-treated samples are represented (B/M in 2 mM, 6 mM, and 12 mM nitrate). Up-regulated and down-regulated transcripts are shown in red and green, respectively.

We performed a functional classification of the DEG in each N-condition (Tables S2–S4) using the Generic Gene Ontology (GO) Term Finder tool (Boyle et al., 2004). This analysis showed a wide range of biological processes that were affected due to fungal infection under different N-conditions (Figure 4). Processes associated with metabolism, both primary and secondary, were significantly affected by the pathogen under all N conditions analyzed (*p* < 0.01). This observation is consistent with previous reports that show that pathogens can reprogram host metabolism, strongly affecting primary, and secondary metabolism in plants (Kliebenstein et al., 2005; Rojas et al., 2014). Figure 4B shows a general overview of metabolic pathways affected by *B. cinerea* infections in each N-condition, using MapMan software (Thimm et al., 2004). Cell wall metabolisms were strongly affected by the fungus infections in plants grown in N-limiting conditions. In contrast, secondary metabolisms were most affected by the fungus infections in N-sufficient conditions. This finding is consistent with the fact that the accumulation of secondary metabolites is a plant defense mechanism triggered by several fungus pathogens (Kliebenstein et al., 2005; Ward et al., 2010; Smith et al., 2014; Pusztahelyi et al., 2015). Interestingly, we found that genes associated with N transporters (NRT2), N and amino acid metabolism were induced in response to *B. cinerea* infection depending on the plant's N-status, with it occurring most in N-sufficient conditions (Figure 4B). In agreement with this observation, it has been shown in a recent report that the expression of several genes involved in N metabolism and its transport are strongly affected in response to infections with *B. cinerea, Phytophthora infestans, Phytophthora parasitica*, and *Pseudomonas syringae* (Fagard et al., 2014). Other biological functions modified by the *B. cinerea* infection, and specifically over-represented under N-sufficient conditions, were transport and oxidation-reduction processes (Figure 4, *p* < 0.01). Redox status significantly impacts both plant defenses and *B. cinerea* infection (Lamb and Dixon, 1997; Torres et al., 2006).

Our enrichment analysis also identified genes that encode proteins previously implicated in defense responses (AbuQamar et al., 2006; Cantu et al., 2009; Windram et al., 2012; Blanco-Ulate et al., 2013). This group of genes was closely examined to evaluate possible correlations between reduced expression of defense-related genes and susceptibility to *B. cinerea* infection. Table 1 shows a subset of these genes induced in plants grown under N-sufficient conditions or repressed under N-limiting conditions. These genes include *anthranilate synthase 1* (*ASA1*), *2-oxophytodienoate reductase 3* (*OPR3*), and *pathogenesis-related 4* (*PR4*). This latter gene encodes a protein with antifungal chitin-binding activity, which is repressed under N-limiting conditions and induced under N-sufficient or higher conditions. In Arabidopsis, this gene has been associated with resistance to necrotrophic pathogens (Catinot et al., 2015) and it is known to be induced by *B. cinerea* infection (AbuQamar et al., 2006). In addition, genes involved in JA and ET response were also identified. The *ACC* oxidase gene involved in ET biosynthesis was significantly induced during fungal infection in plants grown under N-sufficient conditions, but not at low nitrate concentrations (Table 1). Thus, a differential response of key defense-related genes under contrasting nitrate concentrations might partially explain the differences in susceptibility to *B. cinerea* in tomato leaves.

**Table 1:** **Expression profile of defense related genes in response to *B. cinerea* infections under different N conditions**.

**ID Number**	**Description**	**Genome ID**	**AGI number**	**Fold change (log**_**2**_**)**		
				**2**	**6**	**12**
**STRESS RESPONSE**						
LesAffx.22491.2.A1_at	Flavonoid 3-hydroxylase	Solyc12g042480.1.1	AT5G07990	0.4	3.4	4.1
LesAffx.50270.1.S1_at	Strictosidine synthase-like	Solyc02g082900.2.1	AT3G51420	1.1	4.2	3.8
LesAffx.50270.2.S1_at	Strictosidine synthase-like	Solyc02g082900.2.1	AT3G51420	0.8	4.1	3.7
Les.3652.1.S1_at	Beta-1 3-glucanase	Solyc10g079860.1.1	AT3G57270	0.7	3.5	3.9
Les.3673.1.S1_at	Cytochrome P450	Solyc12g042480.1.1	AT4G36220	−1.4	1.5	2.9
Les.4966.1.S1_at	Response to stress	Solyc09g075070.2.1	AT1G02850	−1.6	1.3	2.2
**DEFENSE RESPONSE**						
Les.3406.1.S1_at	Chitinase	Solyc10g055800.1.1	AT3G12500	−0.2	2.8	2.7
Les.248.1.S1_a_at	Pathogenesis-related 4	Solyc01g097270.2.1	AT3G04720	−0.4	2.7	2.7
Les.3683.1.S1_at	Thaumatin, pathogenesis-related	Solyc08g080620.1.1	AT4G11650	−1.1	1.1	2.5
Les.5035.1.S1_at	LRR receptor-like	Solyc01g005730.2.1	AT1G47890	0	1.9	2.5
Les.22.1.S1_at	Oxophytodienoate reductase 3 (OPR3)	Solyc10g086220.1.1	AT1G76690	−1.4	0.4	0.3
**OXIDATION REDUCTION PROCESSES**						
LesAffx.71628.1.S1_at	Thioredoxin superfamily protein	Solyc03g112770.2.1	AT5G63030	−0.3	0.4	0.4
Les.3172.1.S1_at	Glutathione S-transferase	Solyc01g102660.2.1	AT2G02390	−0.4	1.1	1.4
Les.2746.2.A1_at	Glutathione S-transferase	Solyc09g011510.2.1	ND	3.1	2.8	0.6
Les.132.1.S1_at	ACC oxidase	Solyc07g049550.2.1	AT1G05010	−2.9	2.2	3.3
LesAffx.5010.2.S1_at	Cytochrome b561	Solyc03g025840.2.1	AT4G25570	−0.7	0.1	0.2
Les.3449.1.S1_at	Glutathione synthetase	Solyc01g098610.2.1	AT5G27380	−2.0	−0.4	0.1
Les.1925.1.A1_at	Glutathione S-transferase	Solyc05g013950.1.1	AT1G19570	−3.1	0.7	0.7
Les.2955.1.A1_at	Glutathione S-transferase	Solyc02g068900.2.1	ND	−0.9	−0.3	0.4
**HORMONE PATHWAY**						
Les.132.1.S1_at	ACO4 ethylene-forming enzym	Solyc07g049550.2.1	AT1G05010	−2.9	2.2	3.3
Les.3465.1.S1_at	Ethylene response regulator	Solyc09g089610.2.1	AT3G23150	0.6	1.2	2.0
Les.3818.1.S1_at	Ethylene responsive transcription factor	Solyc09g089930.1.1	AT3G23240	−0.7	2.6	2.2
LesAffx.65348.1.S1_at	Anthranilate synthase	Solyc06g006100.2.1	AT5G05730	−0.6	−0.1	0.8
Les.3632.1.S1_at	Lipoxygenase family protein 4 (LOX4)	Solyc03g122340.2.1	AT1G72520	3.5	2.6	0.6

^*^Fold change in gene expression for each gene upon B. cinerea infection are referred to mock-treated samples grown under the same N condition (2, 6, and 12 mM nitrate).

### Network analysis identifies *B. cinerea*-responsive regulatory pathways associated with the plant N status

**Figure 5:**
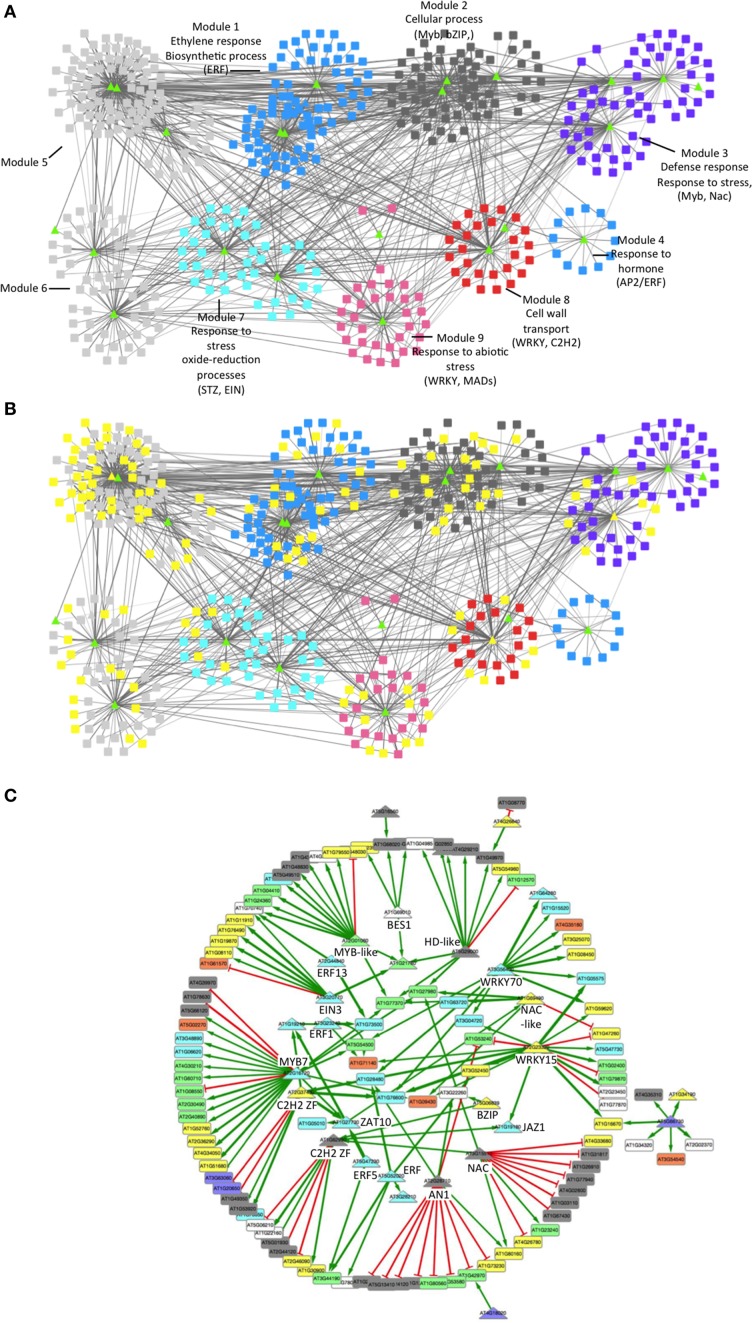
**Network analysis of transcriptomics data predicts a *B. cinerea*-responsive regulatory gene network affected by the plant N-status. (A)** Network analysis of genes with significant N/B ANOVA model. Genes are presented as triangles (TFs) and rectangles (target genes). Colors are used to distinguish each gene network module grouped by topology. The most overrepresented GO term (biological process) and TF in each module are indicated. **(B)** Network analysis of genes with significant N/B ANOVA model in which genes also present in the B ANOVA model are highlighted. Yellow nodes indicate genes that also respond to the infection alone, irrespective of the N concentration. X and Y axes do not represent any particular scale. **(C)** Subnetwork of TFs and their putative targets derived from most co-expressed genes. Arrowhead green lines indicate predicted transcriptional activation, while red lines indicate transcriptional repression. Nodes are color-coded based on biological processes: metabolism (gray), unknown processes (white), defense, and stress response (yellow), response to hormone (purple), JA/ET processes (light blue), oxidative-reduction processes (green), and transport (orange).

To identify functional relationships and key regulatory TFs among the N/B responsive genes, we performed a gene co-expression network analysis. Due to the limited knowledge of gene interactions in tomato, we decided to incorporate data available from *A. thaliana*. For ortholog group assignment, tomato sequences (gene models ITAG release 2.4, https://solgenomics.net), matching probe sets on the GeneChip Tomato Genome array, were aligned to the Arabidopsis genome using OrthoMCL (Li et al., 2003). For network inference, we used all Arabidopsis loci identifiers to predict putative protein-DNA interactions, making use of sequence preference information recently obtained for a large number of Arabidopsis TFs (Weirauch et al., 2014) and the gene co-expression database (Obayashi et al., 2014). As shown in Figure 5A, a network comprising 477 genes was obtained. Genes are represented as nodes connected by edges that depict predicted regulatory interactions. In this analysis, 9 co-expression modules were identified using the community cluster (Glay) algorithm in ClusterMaker tools (Morris et al., 2011). This algorithm recognizes functionally related groups and finds densely connected regions in a network (Su et al., 2010). In addition, we found statistically overrepresented biological functions in 7 of these modules using a hypergeometric test in BINGO tools (adjusted *p* < 0.05 by FDR). Interestingly, network includes functions important for plant defense against necrotrophic fungi, such as stress response, oxide-reduction processes and cell wall and wax biosynthesis processes. Moreover, genes related to ET signaling and the responses to this hormone were also identified as overrepresented in these modules, consistent with the finding describing how ET responses are important for *B. cinerea* resistance in tomato leaves (Díaz et al., 2002). In addition, we found several TFs belonging to the ERF (Ethylene Response Factor) family, which have been implicated in the plant defense response to *B. cinerea* (AbuQamar et al., 2006; Windram et al., 2012). Nevertheless, susceptibility of tomato fruit to *B. cinerea* had been also associated in part with ET pathways (Cantu et al., 2009; Blanco-Ulate et al., 2013), suggesting complex and tissue-specific ET regulatory networks in response to fungus infections.

In order to distinguish the effect of the plant's N-status in the defense response against *B. cinerea* (Figure 5A; N/B ANOVA model) from the general plant response to fungal infection irrespective of its N condition (B ANOVA model), we highlight genes with a significant B (*B. cinerea*) ANOVA model within the mentioned network (Figure 5B). When Figures 5A,B were compared, we noticed that only 40% of the mentioned N/B network responded to the infection as a single (B) factor, supporting the link between fungus infection and N response. Interestingly, the majority of the TFs present in the network also link to this interaction (in Figure 5B, note that most TFs at the center of each module are not highlighted in yellow).

To identify TFs that may control relevant functions in the N status-dependent response to *B. cinerea*, we focused on TFs and those putative targets possessing significant co-expression (Figure 5C). We applied a 90th percentile cut-off in co-expression networks considering the absolute value, such that only the highest scoring 10% of edges were selected. A total of 28 TFs are present in the N/B network, from which 22 were highly connected within its center and might represent important regulators of the N/B interaction. Interestingly, TFs within the network and their respective putative targets are mostly involved in transport, oxide-reduction processes and stress and defense responses. In addition, within this group of regulatory proteins, we also identified overrepresented biological functions related to the response to ET and JA.

### The plant N status modulates the expression of TFs belonging to families involved in plant defense

**Figure 6:**
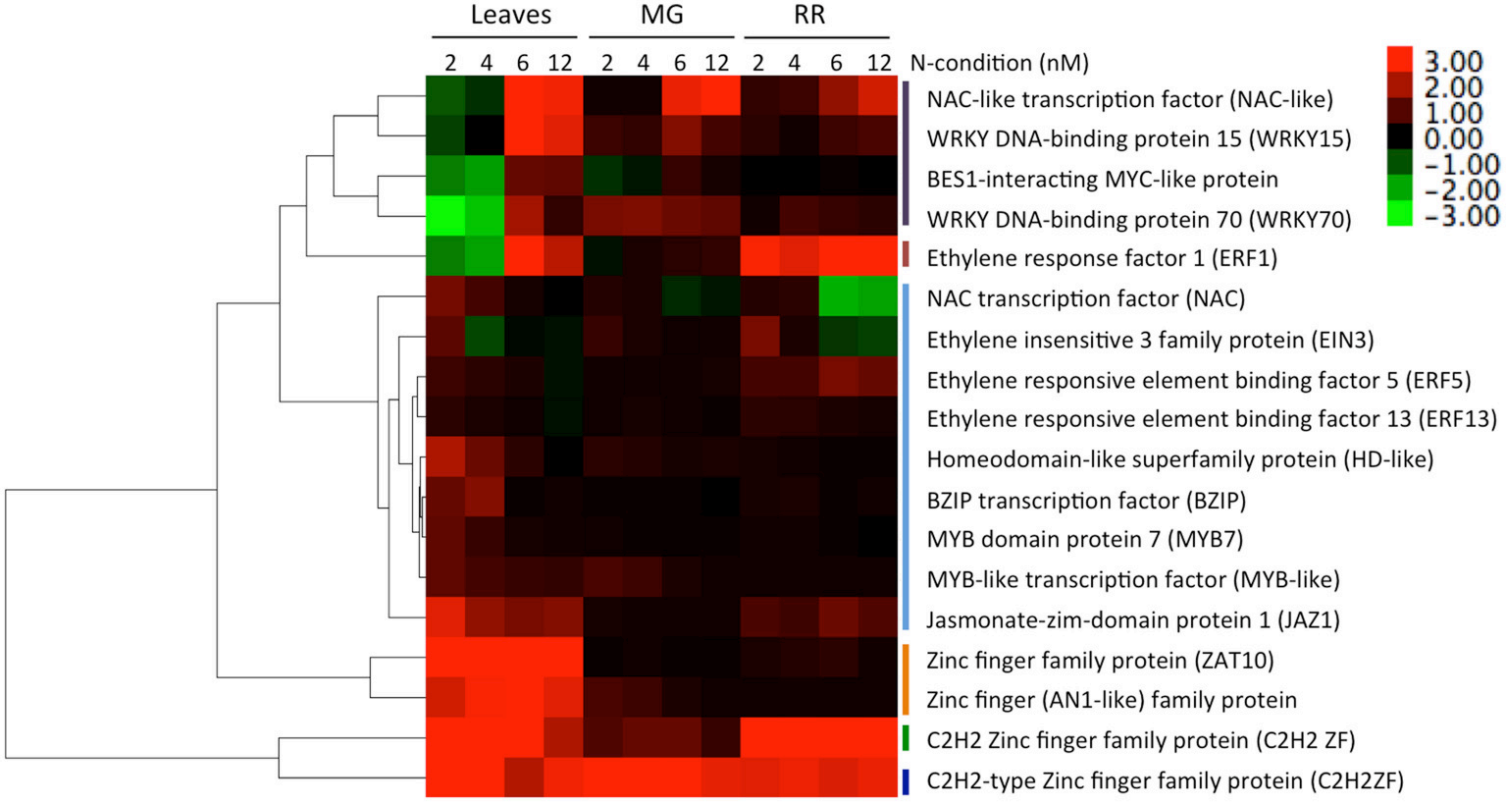
**Hierarchical clustering of mRNA levels for selected TFs showing a differential gene expression pattern in response to the fungus infection under different N conditions**. The dendrogram and colored image were produced using gene expression data after RT-qPCR (*n* = 3). The color scale ranges from saturated green for log_2_ ratios −3.0 and below, to saturated red for log_2_ ratios +3.0 and above. Fold change in gene expression for each gene upon *B. cinerea* infection are referred to mock-treated samples. Each TF is represented by a single row of colored boxes. Six clusters were identified, and are numbered and denoted with a vertical line (from top to bottom) as follows: cluster #1 in gray, #2 in brown, #3 in light blue, #4 in orange, #5 in green, and #6 in blue.

The results depicted in Figure 5 support the importance of plant N metabolism in plant defense pathways. To validate this prediction, we analyzed the expression levels of 18 TF-encoding genes in leaves or fruits infected by *B. cinerea*, from tomato plants grown under all nitrate concentrations used throughout this work (Table S5). An RT-qPCR analysis of selected TFs supported microarray results and network analysis (Figure 6). Hierarchical clustering analysis of the RT-qPCR data revealed 6 clusters. Cluster #1 (Figure 6, in gray), composed of four TF-encoding genes (NAC-like, WRKY15, BIM, and WRKY70), groups genes that are induced by fungal infection under N-sufficient conditions, but repressed in leaves or marginally induced in fruits of plants grown under N-limiting conditions. In Arabidopsis, WRKY 15 and WRKY 70 (Figure 5, Modules 3 and 8, respectively) have been reported as induced when infected with *B. cinerea*. Interestingly, *wrky70* mutant plants showed enhanced susceptibility to this fungus, in a SA dependent manner (AbuQamar et al., 2006).

*B. cinerea* also triggered the induction of zinc-finger TFs (ZAT) genes in leaves, under all N-conditions (Figure 5, Module 7; Figure 6, Cluster #4). ZAT genes are induced in abiotic stress conditions and are required for cytosolic ascorbate peroxidase expression during oxidative stress in Arabidopsis (Rizhsky et al., 2004). These regulatory genes may be involved in the oxidative burst triggered by the pathogen or in oxidation-reduction processes to protect the plant.

Infection by *B. cinerea* enhanced the expression of several TFs involved in the ET response (EIN3, ERF5, and ERF13; Figure 5, Module 1; Figure 6, Cluster #3). ERF5 was slightly induced by the fungal infection, most likely in RR fruits. Among the TFs associated with the ET response, several TFs belong to the ERF family, have been associated with stress responses (McGrath et al., 2005) and as modulators of the SA/JA crosstalk (Van der Does et al., 2013). Interestingly, in infected leaves, ERF1 (Ethylene Response Factor 1; Figure 6, Cluster #2) was significantly induced in N-sufficient conditions and repressed in low N conditions. ERF1 has been implicated in JA and ET signaling, and pathogen defense (Berrocal-Lobo et al., 2002; Lorenzo et al., 2003). In addition, the Jasmonate-ZIM-Domain 1 gene (*JAZ1*; Figure 5, Module 5; Figure 6, Cluster #3) was also induced by the fungus infection in leaves under all analyzed N-conditions. This gene is involved in JA-signaling and, recently, its role in plant defense has been described (Li et al., 2014). These different patterns of expression of TFs related with hormone responses, suggest the existence of several regulatory pathways interacting in a complex and highly dynamic manner, linking plant N status, and the plant defense response.

## Discussion

Although, different agronomic reports indicate that the amount of N fertilization can modulate disease severity caused by pathogens in numerous plant species (Snoeijers et al., 2000; Walters and Bingham, 2007; Dordas, 2008; Fagard et al., 2014), the underlying mechanisms are poorly characterized. In general terms, high N supply decreases the severity of the infections from necrotrophic pathogens, and N-limiting conditions decrease plant susceptibility to biotrophy or hemibiotrophic microorganisms (Walters and Bingham, 2007; Dordas, 2008). Despite these general trends, several reports show contradictory evidence, suggesting a complex relationship linking N metabolism and disease infection processes. In this report, we investigated the role of plant N nutrition in the outcome of the plant-pathogen interaction, employing two important agronomic models: *B. cinerea* and *S. lycopersicum*.

We showed that tomato plants grown under contrasting nitrate conditions exhibit differential susceptibility to fungal infection. We used nitrate as a nitrogen source, since it is the most important N source for plants in agricultural soils (von Wirén et al., 2000) and it plays an important role as the major nitrogen resource in tomato plants (Wang et al., 2001; Fu et al., 2015). In agreement with previous studies, tomato plants grown under N-sufficient or higher conditions showed a significantly decreased susceptibility to *B. cinerea* (Hoffland et al., 1999; Lecompte et al., 2010). We extended previous results to include fruits and showed that there is reduced susceptibility to *B. cinerea* in fruits at two developmental stages (MG and RR) from plants grown under N-sufficient conditions, suggesting that the nitrogen/disease connection might extend beyond vegetative tissues.

The induction of the plant defense response is an energetically costly process (Berger et al., 2007). As such, different studies have suggested that the major role of the plant's primary metabolism during the plant-pathogen interaction is to support the increased energy requirements brought about by the defense response (Bolton, 2009; Kangasjärvi et al., 2012), and some pathogens in early steps of infections trigger a nutrient-limiting environment (Mathioni et al., 2011). In this scenario, it is reasonable to hypothesize that “well nourished” plants may defend better, as observed in tomato grown under N-sufficient conditions. This observation sharply differs from crop plants that present less susceptibility under low nitrogen availability (Davidson et al., 2007; Lecompte et al., 2013). Notably, as observed from our global gene expression profiling, plant metabolic processes were significantly affected by *B. cinerea*, irrespective of the nitrogen condition used to grow the plants. This finding is consistent with previous reports showing that the plant-pathogen interaction regulates plant metabolism (Ward et al., 2010; Rojas et al., 2014). In order to establish a favorable energy balance for defense, it has been suggested that the up-regulation of defense-related genes is compensated by the down-regulation of genes involved in other metabolic pathways (Berger et al., 2007; Massad et al., 2012). In agreement with what has been reported for *B. cinerea* infections in *A. thaliana* (AbuQamar et al., 2006; Windram et al., 2012), several genes involved in photosynthesis were down regulated in our tomato experiments in response to the fungus infection, under all N conditions.

In the context of plant-pathogens interactions, the nutrition of the plant can have an effect on secondary metabolite production. Plant N-status modifies amino acid accumulation involved in the biosynthesis of defense-associated secondary metabolites, such as flavonoids and phenylpropanoids (Ward et al., 2010; Fagard et al., 2014). Phenylpropanoid pathways regulate the resistance against *B. cinerea* in the *sitiens* tomato mutant (Seifi et al., 2013). Accordingly, we found several genes involved in this pathway up-regulated by the fungus infections in N-sufficient conditions. In addition, the amino acid content and its relative concentration are modified during the plant disease process, suggesting that amino acid metabolism can impact plant-pathogen interactions. Recently, reports show that genes involved in amino acid metabolism play a role in plant defense responses to pathogens (Hwang et al., 2011; Seifi et al., 2014). N-metabolism and amino acid homeostasis can modulate the plant redox status affecting the plant disease response (Liu et al., 2010). These results suggest that N metabolism could modulate -at least in part- plant defense responses affecting cellular redox status as well as secondary metabolite pathways.

The relevance of N and amino acid metabolism has been highlighted before in the context of plant-pathogen interactions (Hwang et al., 2011). For instance, several studies have shown that N-related gene expression is altered by pathogens (Pageau et al., 2006; Masclaux-Daubresse and Daniel-Vedele, 2010). Interestingly, the Arabidopsis *lht1* (lysine histidine transport 1) mutant is more resistant to a large spectrum of pathogens, an observation that is associated with an altered redox status (Liu et al., 2010). In this regard, we found several genes associated with redox status, N-metabolism and transporters affected by *B. cinerea* infections, depending on plant N-conditions. NRT2, a putative high-affinity nitrate transporter, was induced only in N-sufficient and higher conditions. Interestingly, in Arabidopsis, two NRT2s have been reported as involved in plant defense (Camañes et al., 2012; Dechorgnat et al., 2012) and the Arabidopsis *nrt2.6* mutant exhibits higher sensitivity to the necrotrophic bacterium *Erwina amylovora*. The susceptibility of this mutant appears to be related to reduced ROS accumulation (Dechorgnat et al., 2012), again linking redox status, N metabolism and defense. Similarly, we found that oxidation-reduction processes were over-represented among the biological functions responding to the fungal infection, under N-sufficient and higher conditions. Recently, nitrate reallocation in plants has been proposed as a regulator of the trade-off between plant growth and environmental adaptation (Zhang et al., 2014). All these results suggest that nitrate metabolism and transport have an impact on the plant's defense response, and that the nitrogen-defense connection goes beyond a direct metabolic relationship. Whether, this can be explained by N-derived alterations in the redox status of the plant requires further investigation.

Different plant hormones may provide an additional layer of regulation, underlying—at least in part—the complex relationship between nitrogen metabolism and plant defense response. It has been described that plant hormones play a role in the defense against *B. cinerea* (AbuQamar et al., 2006; Windram et al., 2012). Specifically, genetic studies on Arabidopsis and tomato indicate that JA and ET are key regulators of this defense response. Treatments with ET in tomato plants increased resistance to this fungus in leaves, while mutants in JA biosynthesis showed increased susceptibility to *B. cinerea* in leaves (Díaz et al., 2002; AbuQamar et al., 2008). Consistent with these prior findings, genes involved in ET response were identified by the GO term analysis in our network of *B. cinerea*-responsive genes associated with the plant N status. Likewise, several TFs associated with the JA/ET response were identified, including ERF1 and ERF5. The latter acts as a positive regulator of JA/ET-responsive defense genes against *B. cinerea* in Arabidopsis (Berrocal-Lobo et al., 2002). Notably, we found that ERF1 was induced in response to *B. cinerea* under N-sufficient conditions, while repressed in plants grown under N-limited ones. This TF has been described as a key regulator of JA/ET signaling, activating plant defense responses against *B. cinerea* in leaves (Berrocal-Lobo et al., 2002; Lorenzo et al., 2003) and necrotrophic *Rhizopus nigricans* in RR tomato fruit (Pan et al., 2013). Moreover, *ERF1* gene expression increases in response to *B. cinerea* infections in MG tomato fruit but is reduced in infected RR fruit (Blanco-Ulate et al., 2013). MG and RR tomato fruits show different susceptibility responses against this fungus infection, each associated with specific expression profiles of genes involved in ET and others hormone biosynthesis (Blanco-Ulate et al., 2013). More studies are necessary to assess the regulation of this TF in response to infections in different tissues.

Although, the plant hormone SA has been associated with the resistance to biotrophic pathogens, its role in plant defense against *B. cinerea* is not completely clear. On the one hand, the exogenous application of SA has been reported to result in decreased susceptibility to this fungus in tomato (Audenaert et al., 2002) and Arabidopsis (Ferrari et al., 2003), suggesting that SA affects the resistance against this fungus. On the other hand, the stearoyl-ACP desaturase (*ssi2*) Arabidopsis mutant, which exhibits increased SA levels and higher expression of the *PR1* gene, displayed, increased susceptibility to *B. cinerea* (Kachroo et al., 2001; Nandi et al., 2005). High resolution temporal transcriptomic analysis of the Arabidopsis defense response against this fungus, revealed complex regulatory networks, with different timings for JA, ET, and SA signaling (Windram et al., 2012) as well as the accumulation of plant phytoalexins and other defense-related proteins and molecules (Van Baarlen et al., 2004; AbuQamar et al., 2006; Scalschi et al., 2015). Our data support a role for these signaling pathways connecting N status and infection. For instance, the silencing of the *OPR3* gene enhances susceptibility to *B. cinerea* by affecting the JA biosynthesis pathway (Scalschi et al., 2015), and we found this gene to be repressed under N-limiting conditions. The WRKY70 TF, which is up-regulated in response to *B. cinerea* infection, plays a role in SA-JA crosstalk, activating SA-induced genes and repressing JA-responsive genes (Li et al., 2004). Interestingly, the *wrky70* mutant is more susceptible to this fungus (AbuQamar et al., 2006), since notably, a gene encoding for a putative WRKY70 TF in tomato is the most repressed gene in response to *B. cinerea*, as shown here, but only under low N availability, a condition in which higher susceptibility to the fungus was observed.

Taken together, the findings reported here suggest that N metabolism affects different mechanisms, including redox status, secondary metabolites and plant hormones (JA and ET), the latter of which globally alter the expression of defense-related genes. In sum, these mechanisms together modulate disease susceptibility and hence the outcome of the plant-pathogen interaction under different N conditions.

## Materials and methods

### Growth conditions of tomato plants

*Solanum lycopersicum* cv. *MicroTom* plants were cultivated under different N growth conditions employing 16:8 h light: dark cycles (150 micromoles/m^2^/s) at 20°C. Plants seeds were spread in plastic pots containing sterilized vermiculite, previously equilibrated with Murashige & Skoog (MS) modified basal salt mixture without nitrogen (PhytoTechnology Laboratories). This nutrient solution was supplemented with different concentrations of nitrate. Six mM nitrate was found to give maximal growth and thus considered as N-sufficient condition, while 2 mM and 4 mM nitrate were used as severe and mild N-limiting conditions, respectively. To evaluate a higher N input, tomato plants were grown using 12 mM nitrate. Four nutrition solutions—each containing the mentioned nitrate concentrations—were prepared and added to each pot every 2 days, employing the same volume (50 ml). Shoot biomass (dry weight) and N concentration (Dumas method) were determined from an average of 8 plants after 4 weeks of growth. Fruits were tagged at 2-day post anthesis (dpa) and harvested at 32 and 40 dpa as mature green and ripening red (MG and RR, respectively). A color chart was employed to confirm ripening stages.

### *Botrytis cinerea* growth conditions and virulence assays

The necrotrophic fungus used in this study was *B. cinerea* strain B05.10. This strain was routinely cultivated in Petri dishes containing potato dextrose agar (PDA, AppliChem) with 10% homogenized bean leaves. Conidia employed for virulence assays were obtained from an agar plug followed by glass-wool filtration using Gamborg B5-2% glucose (Duchefa Biochemie) medium.

For virulence assays, 4-week-old tomato plants grown under different nitrate conditions were inoculated in the third and fourth fully expanded leaves *in planta* as described (Cantu et al., 2008; Canessa et al., 2013). Briefly, conidia were suspended in Gamborg B5-2% glucose medium and adjusted to a final concentration of 5 × 10^5^ conidia/ml in the same medium supplemented with 10 mM KH_2_PO_4_/K_2_HPO_4_, pH 6.4. Conidial suspensions (10 μl) were used to inoculate leaves of six tomato plants obtained from each N condition analyzed. All plants were incubated inside plastic boxes at 20°C employing the growth conditions mentioned above, under humid environment for the indicated periods of time. Lesions were measured using the ImageJ software employing an external calibration scale (Schneider et al., 2012). On the other hand, fruits were inoculated at the day of harvest, as described (Cantu et al., 2008; Canessa et al., 2013). Briefly, fruits were first disinfected using 10% (v/v) bleach, followed by three consecutive water rinses. Thereafter, fruits were carefully punctured (2 mm depth, 1 mm diameter) employing a sterile needle at five sites per tomato. Subsequently, fruits were inoculated with 10 μl of conidia suspension (5 × 10^5^ conidia/ml). Tomatoes used as mock material were inoculated with the same volume of Gamborg B5-2% glucose-10 mM KH_2_PO_4_/K_2_HPO_4_ solution. All fruit samples were incubated at 20°C in high humidity, during the indicated periods of time (3, 4, and 5 dpi). At the end of the infection period, tomato lesions were measured as mentioned above. The evaluation of tomato susceptibility to *B. cinerea* was independently performed three times employing at least 4 fruits for each experimental condition.

For quantification of fungal development on plant tissue, DNA from leaves or fruits for each experimental condition was isolated following a method for genomic DNA described by Edwards et al. (1991). For real time PCR quantification, 25 μl samples were prepared containing 10 μl of DNA solutions and 300 nM of appropriate primers. Real-time PCR reactions were run in triplicates using the Brilliant SYBR Green QPCR Reagents on a StepOnePlus™ Real-Time PCR System (Life Technologies), as described bellow in RT-qPCR section. The sequences of the primers used in this study are detailed in Table S6. Serial dilutions of pure genomic DNA from each species were used to develop a calibration curve, as described (Gachon and Saindrenan, 2004).

### RNA isolation

Total RNA was extracted from both mock-treated and fungus-infected tomato leaves and fruits, following a CTAB-spermidine extraction protocol (Reid et al., 2006). In the case of leaves, a total of 12 leaves per N treatment were pulled together. This procedure was repeated three times (*n* = 3) and further used in microarray analysis and RT-qPCR experiments (see below). For fruits, total RNA was prepared from the combined fruit outer pericarp and epidermis, within approximately 0.25 cm radius around the *B. cinerea* inoculation sites. Each biological replicate (*n* = 3) consisted of an independent pool of samples from four different fruits. RNA samples were further quantified and analyzed by microfluidic analysis employing the Agilent Technologies' 2100 Bioanalyzer, following the manufacturer's instructions.

### Microarray analysis

For microarray hybridizations, three mock-inoculated and three fungus-infected biological replicates from tomato plants grown under 2, 6, and 12 mM nitrate were selected for global gene expression analysis. Five hundred nanograms of total RNA was processed for microarray hybridization using the GeneChip one-cycle target-labeling kit (Affymetrix), according to the manufacturer's instructions. The fragmented aRNA (7.5 μg) was hybridized on a GeneChip Tomato genome array (containing 10038 tomato probe sets for more than 9200 tomato genes) using standard procedures (45°C for 16 h). The arrays were washed and stained in a Fluidics Station 450 (Affymetrix). Array scanning was carried out with the GeneChip scanner 300 and image analysis was performed using the GeneChip Operating Software. Thereafter, GeneChip array data were quality assessed using a set of standard quality control steps described in the Affymetrix manual “GeneChip Expression Analysis: Data Analysis Fundamentals.” Array data were processed and normalized with RMA (Robust Multi-Array Average) (Irizarry et al., 2003) using the affy R package (Gautier et al., 2004). Approximately 60–75% of probe sets were significantly detected in all microarray hybridizations. To evaluate array reproducibility, spearman rank coefficients were computed and ranged between 0.97 and 0.99. The raw data for all hybridizations can be found in NCBIs Gene Expression Omnibus (Edgar et al., 2002) and are accessible through GEO Series accession number GSE73006.

To determine DEG in response to *B. cinerea* infection affected by the N-conditions, normalized data were subjected to a Two-way analysis of variance (ANOVA) with adjusted *p* < 0.01 by Benjamini and Hochberg false discovery rate correction (Benjamini and Hochberg, 1995). For ANOVA analysis, we used a model with expression Y of a given gene i calculated as Y_*i*_= β_0_ + β_1_B + β_2_N + β_3_N/B + ε, where β_0_ is the global mean, and where β_1_, β_2_, and β_3_ are the effects of the *B. cinerea* infection, the N-condition and the interaction between these two factors (N/B), respectively (Table S1). The variable ε corresponds to the unexplained variance. In addition, to identify DEG between two conditions (e.g., infected and control samples at each N-condition) the Rank-Product method was used (*p* < 0.05) (Breitling et al., 2004; see Tables S2–S4).

### Functional annotation of differentially expressed genes (DEG)

For the assignment of functional annotations and categorizations for DEG, we employed the easy to use web tool Generic Gene Ontology (GO) Term Finder (Boyle et al., 2004). For this purpose, we constructed a custom-made GO database employing InterProScan 5 (Jones et al., 2014) and all predicted protein coding genes in the *S. lycopersicum* genome database (gene models ITAG release 2.4, https://solgenomics.net).

### Gene network analysis

Genes possessing a significant Nitrogen/*B. cinerea* (N/B) interaction factor were selected to carry out a gene network construction (Table S1). First, we perform an analysis of ortholog group assignment between tomato and *A. thaliana*. For this purpose, tomato sequences (gene models ITAG release 2.4), matching to probe sets on the array were aligned to the Arabidopsis genome, using OrthoMCL (Li et al., 2003) orthologs genes were assigned. Thereafter, with all Arabidopsis loci identifiers, a gene co-expression network was constructed using 11,171 microarray experiments obtained from the ATTED-II database (Obayashi et al., 2014), accessed in March 2015. Co-expression values were calculated using weighted Pearson's correlation coefficient, as described in Obayashi et al. (2014). This database collects gene expression data in Arabidopsis from a wide range of microarray experiments. We included protein–DNA interactions (Weirauch et al., 2014), considering at least one TF binding site in the upstream gene region (1000 bp) and over-representation of the TF binding site (two standard deviations) above the mean occurrence in all the upstream sequences in the genome. To improve the regulatory interaction predictions, the protein–DNA interactions were filtered to include only transcription factor/target pairs for which co-expression values are highly (cut-off above the 90th percentile considering absolute value of gene co-expression) (Obayashi et al., 2014) and significantly correlated (*p* < 0.05) in our microarray experiment. The resulting network was visualized using CYTOSCAPE (Data Sheet S1; Shannon et al., 2003).

### RT-qPCR and clustering analysis of gene expression data

cDNA synthesis was carried out using the Improm-II reverse transcriptase according to the manufacturer's instructions (Promega). RT-qPCR was carried out using the Brilliant SYBR Green QPCR Reagents on a StepOnePlus™ Real-Time PCR System (Life Technologies). For mRNA levels normalization, the *PROTEIN PHOSPHATASE 2A catalytic subunit (PP2Acs, SGN-U567355)* was used (Løvdal and Lillo, 2009; Dekkers et al., 2012). The expression of this reference gene was stable in our microarray data (*CV* = 2%) and in microarrays in response to *B. cinerea* in tomato fruit at two developmental stages (MG and RR, *CV* = 3% Cantu et al., 2009).

Total RT-qPCR reaction volume was 25 μl, containing 2.0 μl of cDNA template and 140 nM of each primer. The reactions were performed under the following conditions: 95°C for 10 min, followed by 40 cycles of 95°C, 30 s; 55°C, 30 s; and 72°C, 30 s, followed by a melting curve analysis from 55 to 95°C. Fluorescence values were acquired during the annealing period of the RT-qPCR procedure. All experiments were performed with three biological replicates, with their corresponding three technical replicates. To determine the mRNA levels in response to the *B. cinerea* infection, infected leaves and fruits from plants grown under 2, 4, 6, and 12 mM nitrate were compared to their corresponding mock-treated control grown under same culture conditions. Genes showing a similar expression pattern were analyzed and visualized using the average-linkage hierarchical clustering performed in Cluster 2.11 software, as described (Eisen et al., 1998).

## Author contributions

PC, GH, IR, AV performed and analyzed virulence assays; GH, AV performed and analyzed microarray experiments; PC and AV conducted the GO analysis of gene expression data; TM, JC, AV conducted bioinformatics analysis for gene network construction; IR, GH, JR, AV, PC completed RT-qPCR analysis; AV, PC, and RG analyzed all data and wrote the paper.

### Conflict of interest statement

The authors declare that the research was conducted in the absence of any commercial or financial relationships that could be construed as a potential conflict of interest.

## Supplementary material

Figure S1 **Tomato plants grown under contrasting N conditions. (A)** Shoots biomass (average) of *MicroTom* tomato plants (4-weeks-old) grown under contrasting N conditions. **(B)** N percentage of plants, determined as dry weight (see Materials and Methods; *n* = 4). Different letters indicate significant differences among treatments (*p* ≤ 0.05; error bars indicate SEM).

Figure S2 **Fungal growth quantification over a *B. cinerea* infection time-course**. The abundance of the fungus was quantified by qPCR amplification of *cutA* (see Materials and Methods) in infected leaves samples **(A)**, and MG **(B)** and RR **(C)** fruits. Different letters indicate significant differences among treatments (*p* ≤ 0.05; error bars indicate SEM; *n* = 3).

Figure S3 **ANOVA analysis of microarray gene expression data**. The number of genes with significant factors obtained by Two-way ANOVA analysis of global expression data is represented (*p* < 0.01), employing the SUNGEAR tool (Poultney et al., 2007). The triangle and its vertices represent the analyzed factors: *B. cinerea* (B), N conditions (N) and the interaction between these two factors (N/B). Circles (and their respective size) represent the number of genes controlled by the different factors, as indicated by the arrows around the circles.

Table S1 **Microarray expression data for genes with N/B ANOVA model (adjusted *p* < 0.01)**. The table includes the Affymetrix probes, the tomato accessions (ITAG2.4, Sol Genomics Network, https://solgenomics.net) and their annotations, Arabidopsis (TAIR, http://arabidopsis.org) or GeneBank accessions, the *p*-value adjusted by FDR and the log2-fold changes of the comparison between *B. cinerea* infected and mock-treated sample (B/M) in each N-conditions (2, 6, and 12 mM nitrate).

Table S2 **Microarray expression data for genes that are either up- or down-regulated by the *B. cinerea* infection in comparison to mock-inoculated plants grown in N-limiting conditions (2 mM nitrate) (ANOVA; Rank-products, *p* < 0.05)**. The table includes the Affymetrix probes, the tomato accessions (ITAG2.4, Sol Genomics Network, https://solgenomics.net) and their annotations, Arabidopsis (TAIR, http://arabidopsis.org) or GeneBank accessions, the *p*-value, Gene Ontology and the log2-fold changes of the comparison between *B. cinerea* infected and mock-treated sample (B/M) grown in N-limiting conditions (2 mM nitrate).

Table S3 **Microarray expression data for genes that are either up- or down-regulated by the *B. cinerea* infection in comparison to mock-inoculated plants grown in N- sufficient conditions (6 mM nitrate) (ANOVA; Rank-products, *p* < 0.05)**. The table includes the Affymetrix probes, the tomato accessions (ITAG2.4, Sol Genomics Network, https://solgenomics.net) and their annotations, Arabidopsis (TAIR, http://arabidopsis.org) or GeneBank accessions, the *p*-value, Gene Ontology and the log2-fold changes of the comparison between *B. cinerea* infected and mock-treated sample (B/M) grown in N-sufficient conditions (6 mM nitrate).

Table S4 **Microarray expression data for genes that are either up- or down-regulated by the *B. cinerea* infection in comparison to mock-inoculated plants grown in N- sufficient conditions (12 mM nitrate) (ANOVA; Rank-products, *p* < 0.05)**. The table includes the Affymetrix probes, the tomato accessions (ITAG2.4, Sol Genomics Network, https://solgenomics.net) and their annotations, Arabidopsis (TAIR, http://arabidopsis.org) or GeneBank accessions, the *p*-value, Gene Ontology, and the log2-fold changes of the comparison between *B. cinerea* infected and mock-treated sample (B/M) grown in N-limiting sufficient (12 mM nitrate).

Table S5 **Candidate TF used for RT-qPCR analysis**. The table includes the Arabidopsis (TAIR, http://arabidopsis.org) and the tomato accessions (Sol Genomics Network, https://solgenomics.net), the gene names, the module in network analysis and changes in relative expression (log2) between infected and mock-treated samples in leaves and fruits (MG and RR) from plants grown under different N-conditions (2 mM, 4 mM, 6 mM, and 12 mM nitrate).

Table S6 **Primer sequences used for qPCR**.

Data Sheet S1 **Gene network in cytoscape**.

## References

[B1] AbuQamar S. Chai M.-F. Luo H. Song F. Mengiste T. (2008). Tomato protein kinase 1b mediates signaling of plant responses to necrotrophic fungi and insect herbivory. Plant Cell 20, 1964–1983. 10.1105/tpc.108.059477 18599583 PMC2518242

[B2] AbuQamar S. Chen X. Dhawan R. Bluhm B. Salmeron J. Lam S.. (2006). Expression profiling and mutant analysis reveals complex regulatory networks involved in Arabidopsis response to Botrytis infection. Plant J. 48, 28–44. 10.1111/j.1365-313X.2006.02849.x 16925600

[B3] Alba R. Payton P. Fei Z. McQuinn R. Debbie P. Martin G. B.. (2005). Transcriptome and selected metabolite analyses reveal multiple points of ethylene control during tomato fruit development. Plant Cell 17, 2954–2965. 10.1105/tpc.105.036053 16243903 PMC1276022

[B4] Audenaert K. Pattery T. Cornelis P. Höfte M. (2002). Induction of systemic resistance to *Botrytis cinerea* in tomato by Pseudomonas aeruginosa 7NSK2: role of salicylic acid, pyochelin, and pyocyanin. Mol. Plant Microbe Interact. 15, 1147–1156. 10.1094/MPMI.2002.15.11.1147 12423020

[B5] Ballini E. Nguyen T. T. Morel J.-B. (2013). Diversity and genetics of nitrogen-induced susceptibility to the blast fungus in rice and wheat. Rice 6:32. 10.1186/1939-8433-6-32 24280346 PMC4883689

[B6] Benjamini Y. Hochberg Y. (1995). Controlling the false discovery rate: a practical and powerful approach to multiple testing. J. R. Statist. Soc B 10.1198/016214504000001907

[B7] Berger S. Sinha A. K. Roitsch T. (2007). Plant physiology meets phytopathology: plant primary metabolism and plant-pathogen interactions. J. Exp. Bot. 58, 4019–4026. 10.1093/jxb/erm298 18182420

[B8] Berrocal-Lobo M. Molina A. Solano R. (2002). Constitutive expression of ETHYLENE-RESPONSE-FACTOR1 in Arabidopsis confers resistance to several necrotrophic fungi. Plant J. 29, 23–32. 10.1046/j.1365-313x.2002.01191.x 12060224

[B9] Blanco-Ulate B. Vincenti E. Powell A. L. T. Cantu D. (2013). Tomato transcriptome and mutant analyses suggest a role for plant stress hormones in the interaction between fruit and *Botrytis cinerea*. Front. Plant Sci. 4:142. 10.3389/fpls.2013.00142 23717322 PMC3653111

[B10] Bolton M. D. (2009). Primary metabolism and plant defense–fuel for the fire. Mol. Plant Microbe Interact. 22, 487–497. 10.1094/MPMI-22-5-0487 19348567

[B11] Boyle E. I. Weng S. Gollub J. Jin H. Botstein D. (2004). GO:: TermFinder—open source software for accessing Gene Ontology information and finding significantly enriched Gene Ontology terms associated with a list of genes. Bioinformatics 20, 3710–3715. 10.1093/bioinformatics/bth456 15297299 PMC3037731

[B12] Breitling R. Armengaud P. Amtmann A. Herzyk P. (2004). Rank products: a simple, yet powerful, new method to detect differentially regulated genes in replicated microarray experiments. FEBS Lett. 573, 83–92. 10.1016/j.febslet.2004.07.055 15327980

[B13] Caarls L. Pieterse C. M. J. Van Wees S. C. M. (2015). How salicylic acid takes transcriptional control over jasmonic acid signaling. Front. Plant Sci. 6:170. 10.3389/fpls.2015.00170 25859250 PMC4373269

[B14] Camañes G. Pastor V. Cerezo M. García-Andrade J. Vicedo B. García-Agustín P.. (2012). A deletion in NRT2.1 attenuates Pseudomonas syringae-induced hormonal perturbation, resulting in primed plant defenses. Plant Physiol. 158, 1054–1066. 10.1104/pp.111.184424 22158760 PMC3271742

[B15] Canessa P. Schumacher J. Hevia M. A. Tudzynski P. Larrondo L. F. (2013). Assessing the effects of light on differentiation and virulence of the plant pathogen *Botrytis cinerea*: characterization of the White Collar Complex. PLoS ONE 8:e84223. 10.1371/journal.pone.0084223 24391918 PMC3877267

[B16] Cantu D. Blanco-Ulate B. Yang L. Labavitch J. M. Bennett A. B. Powell A. L. T. (2009). Ripening-regulated susceptibility of tomato fruit to *botrytis cinerea* requires NOR but not RIN or Ethylene. Plant Physiol. 150, 1434–1449. 10.1104/pp.109.138701 19465579 PMC2705034

[B17] Cantu D. Vicente A. R. Greve L. C. Dewey F. M. Bennett A. B. Labavitch J. M.. (2008). The intersection between cell wall disassembly, ripening, and fruit susceptibility to *Botrytis cinerea*. Proc. Natl. Acad. Sci. U.S.A. 105, 859–864. 10.1073/pnas.0709813105 18199833 PMC2242701

[B18] Castro Marín I. Loef I. Bartetzko L. Searle I. Coupland G. Stitt M.. (2010). Nitrate regulates floral induction in Arabidopsis, acting independently of light, gibberellin and autonomous pathways. Planta 233, 539–552. 10.1007/s00425-010-1316-5 21113723 PMC3043248

[B19] Catinot J. Huang J. B. Huang P. Y. (2015). ETHYLENE RESPONSE FACTOR 96 positively regulates Arabidopsis resistance to necrotrophic pathogens by direct binding to GCC elements of jasmonate–and …. Plant. 10.1111/pce.12583 26038230

[B20] Davidson J. A. Pande S. Bretag T. W. Lindbeck K. D. Krishna-Kishore G. (2007). Biology and management of botrytis spp. in legume crops, in Botrytis: Biology, Pathology and Control, eds Elad Y. Williamson B. Tudzynski P. Delen N. (Dordrecht: Springer), 295–318. 10.1007/978-1-4020-2626-3_16

[B21] Dean R. van Kan J. A. L. Pretorius Z. A. Hammond-Kosack K. E. Di Pietro A. Spanu P. D.. (2012). The Top 10 fungal pathogens in molecular plant pathology. Mol. Plant Pathol. 13, 414–430. 10.1111/J.1364-3703.2011.00783.X 22471698 PMC6638784

[B22] Dechorgnat J. Patrit O. Krapp A. Fagard M. Daniel-Vedele F. (2012). Characterization of the Nrt2.6 gene in Arabidopsis thaliana: a link with plant response to biotic and abiotic stress. PLoS ONE 7:e42491. 10.1371/journal.pone.0042491 22880003 PMC3413667

[B23] Dekkers B. J. W. Willems L. Bassel G. W. van Bolderen-Veldkamp R. P. M. Ligterink W. Hilhorst H. W. M.. (2012). Identification of reference genes for RT-qPCR expression analysis in Arabidopsis and tomato seeds. Plant Cell Physiol. 53, 28–37. 10.1093/pcp/pcr113 21852359

[B24] Díaz J. ten Have A. van Kan J. A. (2002). The role of ethylene and wound signaling in resistance of tomato to *Botrytis cinerea*. Plant Physiol. 129, 1341–1351. 10.1104/pp.001453 12114587 PMC166527

[B25] Dietrich R. Plo ß. K. Heil M. (2004). Constitutive and induced resistance to pathogens in Arabidopsis thaliana depends on nitrogen supply. Plant Cell Environ. 27, 896–906. 10.1111/j.1365-3040.2004.01195.x

[B26] Dordas C. (2008). Role of nutrients in controlling plant diseases in sustainable agriculture. A Rev. Agron. Sustain. Dev. 28, 33–46. 10.1051/agro:2007051

[B27] Edgar R. Domrachev M. Lash A. E. (2002). Gene Expression Omnibus: NCBI gene expression and hybridization array data repository. Nucleic Acids Res. 30, 207–210. 10.1093/nar/30.1.207 11752295 PMC99122

[B28] Edwards K. Johnstone C. Thompson C. (1991). A simple and rapid method for the preparation of plant genomic DNA for PCR analysis. Nucleic Acids Res. 19, 1349. 2030957 10.1093/nar/19.6.1349 PMC333874

[B29] Eisen M. B. Spellman P. T. Brown P. O. Botstein D. (1998). Cluster analysis and display of genome-wide expression patterns. Proc. Natl. Acad. Sci. U.S.A. 95, 14863–14868 9843981 10.1073/pnas.95.25.14863 PMC24541

[B30] Fagard M. Launay A. Clément G. Courtial J. Dellagi A. Farjad M.. (2014). Nitrogen metabolism meets phytopathology. J. Exp. Bot. 65, 5643–5656. 10.1093/jxb/eru323 25080088

[B31] Farmer E. E. Alméras E. Krishnamurthy V. (2003). Jasmonates and related oxylipins in plant responses to pathogenesis and herbivory. Curr. Opin. Plant Biol. 6, 372–378. 10.1016/S1369-5266(03)00045-1 12873533

[B32] Ferrari S. Plotnikova J. M. De Lorenzo G. (2003). Arabidopsis local resistance to *Botrytis cinerea* involves salicylic acid and camalexin and requires EDS4 and PAD2, but not SID2, EDS5 or PAD4. Plant J. 35, 193–205. 10.1046/j.1365-313X.2003.01794.x 12848825

[B33] Fu Y. Yi H. Bao J. Gong J. (2015). LeNRT2.3 functions in nitrate acquisition and long-distance transport in tomato. FEBS Lett. 589, 1072–1079. 10.1016/j.febslet.2015.03.016 25819437

[B34] Gachon C. Saindrenan P. (2004). Real-time PCR monitoring of fungal development in Arabidopsis thaliana infected by Alternaria brassicicola and *Botrytis cinerea*. Plant Physiol. Biochem. 42, 367–371. 10.1016/j.plaphy.2004.04.001 15191738

[B35] Gautier L. Cope L. Bolstad B. M. Irizarry R. A. (2004). affy—analysis of Affymetrix GeneChip data at the probe level. Bioinformatics 20, 307–315. 10.1093/bioinformatics/btg405 14960456

[B36] Giovannoni J. J. (2007). Fruit ripening mutants yield insights into ripening control. Curr. Opin. Plant Biol. 10, 283–289. 10.1016/j.pbi.2007.04.008 17442612

[B37] Glazebrook J. (2005). Contrasting mechanisms of defense against biotrophic and necrotrophic pathogens. Annu. Rev. Phytopathol. 43, 205–227. 10.1146/annurev.phyto.43.040204.135923 16078883

[B38] Good A. G. Shrawat A. K. Muench D. G. (2004). Can less yield more? Is reducing nutrient input into the environment compatible with maintaining crop production? Trends Plant Sci. 9, 597–605. 10.1016/j.tplants.2004.10.008 15564127

[B39] Hey S. J. Byrne E. Halford N. G. (2010). The interface between metabolic and stress signalling. Ann. Bot. 105, 197–203. 10.1093/aob/mcp285 20007158 PMC2814758

[B40] Hirel B. Tétu T. Lea P. J. Dubois F. (2011). Improving nitrogen use efficiency in crops for sustainable agriculture. Sustainability 3, 1452–1485. 10.3390/su3091452

[B41] Hoffland E. Jeger M. J. van Beusichem M. L. (2000). Effect of nitrogen supply rate on disease resistance in tomato depends on the pathogen. Plant Soil 218, 239–247. 10.1023/A:1014960507981

[B42] Hoffland E. van Beusichem M. L. Jeger M. J. (1999). Nitrogen availability and susceptibility of tomato leaves to *Botrytis cinerea*. Plant Soil 210, 263–272. 10.1023/A:1004661913224

[B43] Hwang I. S. An S. H. Hwang B. K. (2011). Pepper asparagine synthetase 1 (CaAS1) is required for plant nitrogen assimilation and defense responses to microbial pathogens. Plant J. 67, 749–762. 10.1111/j.1365-313X.2011.04622.x 21535260

[B44] Irizarry R. A. Hobbs B. Collin F. Beazer-Barclay Y. D. Antonellis K. J. Scherf U.. (2003). Exploration, normalization, and summaries of high density oligonucleotide array probe level data. Biostat. 4, 249–264. 10.1093/biostatistics/4.2.249 12925520

[B45] Jones P. Binns D. Chang H.-Y. Fraser M. Li W. McAnulla C.. (2014). InterProScan 5: genome-scale protein function classification. Bioinformatics 30, 1236–1240. 10.1093/bioinformatics/btu031 24451626 PMC3998142

[B46] Kachroo P. Shanklin J. Shah J. Whittle E. J. Klessig D. F. (2001). A fatty acid desaturase modulates the activation of defense signaling pathways in plants. Proc. Natl. Acad. Sci. U.S.A. 98, 9448–9453. 10.1073/pnas.151258398 11481500 PMC55441

[B47] Kangasjärvi S. Neukermans J. Li S. Aro E.-M. Noctor G. (2012). Photosynthesis, photorespiration, and light signalling in defence responses. J. Exp. Bot. 63, 1619–1636. 10.1093/jxb/err402 22282535

[B48] Kliebenstein D. J. Rowe H. C. Denby K. J. (2005). Secondary metabolites influence Arabidopsis/Botrytis interactions: variation in host production and pathogen sensitivity. Plant J. 44, 25–36. 10.1111/j.1365-313X.2005.02508.x 16167893

[B49] Lamb C. Dixon R. A. (1997). The oxidative burst in plant disease resistance. Annu. Rev. Plant Physiol. Plant Mol. Biol. 48, 251–275. 10.1146/annurev.arplant.48.1.251 15012264

[B50] Lau G. Hamer J. E. (1996). Regulatory genes controlling MPG1 expression and pathogenicity in the rice blast fungus magnaporthe grisea. Plant Cell 8, 771–781. 10.1105/tpc.8.5.771 12239399 PMC161136

[B51] Lecompte F. Abro M. A. Nicot P. C. (2010). Contrasted responses of *Botrytis cinerea* isolates developing on tomato plants grown under different nitrogen nutrition regimes. Plant Pathol. 59, 891–899. 10.1111/j.1365-3059.2010.02320.x

[B52] Lecompte F. Abro M. A. Nicot P. C. (2013). Can plant sugars mediate the effect of nitrogen fertilization on lettuce susceptibility to two necrotrophic pathogens: *Botrytis cinerea* and Sclerotinia sclerotiorum? Plant Soil 369, 387–401. 10.1007/s11104-012-1577-9

[B53] Li C. He X. Luo X. Xu L. Liu L. Min L.. (2014). Cotton WRKY1 mediates the plant defense-to-development transition during infection of cotton by Verticillium dahliae by activating JASMONATE ZIM-DOMAIN1 expression. Plant Physiol. 166, 2179–2194. 10.1104/pp.114.246694 25301887 PMC4256851

[B54] Li J. Brader G. Palva E. T. (2004). The WRKY70 transcription factor: a node of convergence for jasmonate-mediated and salicylate-mediated signals in plant defense. Plant Cell 16, 319–331. 10.1105/tpc.016980 14742872 PMC341906

[B55] Li L. Stoeckert C. J. Jr. Roos D. S. (2003). OrthoMCL: identification of ortholog groups for eukaryotic genomes. Genome Res. 13, 2178–2189. 10.1101/gr.1224503 12952885 PMC403725

[B56] Linquist B. A. Byous E. Jones G. Williams J. F. Six J. (2008). Nitrogen and potassium fertility impacts on aggregate sheath spot disease and yields of rice. Plant Prod. Sci. 11, 260–267. 10.1626/pps.11.260

[B57] Liu G. Ji Y. Bhuiyan N. H. Pilot G. Selvaraj G. Zou J.. (2010). Amino acid homeostasis modulates salicylic acid-associated redox status and defense responses in Arabidopsis. Plant Cell 22, 3845–3863. 10.1105/tpc.110.079392 21097712 PMC3015111

[B58] Long D. H. Lee F. N. TeBeest D. O. (2007). Effect of nitrogen fertilization on disease progress of rice blast on susceptible and resistant cultivars. Plant Dis. 84, 403–409. 10.1094/PDIS.2000.84.4.403 30841161

[B59] Lorenzo O. Piqueras R. Sánchez-Serrano J. J. Solano R. (2003). ETHYLENE RESPONSE FACTOR1 integrates signals from ethylene and jasmonate pathways in plant defense. Plant Cell 15, 165–178. 10.1105/tpc.007468 12509529 PMC143489

[B60] Løvdal T. Lillo C. (2009). Reference gene selection for quantitative real-time PCR normalization in tomato subjected to nitrogen, cold, and light stress. Anal. Biochem. 387, 238–242. 10.1016/j.ab.2009.01.024 19454243

[B61] Masclaux-Daubresse C. Daniel-Vedele F. (2010). Nitrogen uptake, assimilation and remobilization in plants: challenges for sustainable and productive agriculture. Ann Bot. 105, 1141–1157. 10.1093/aob/mcq028 20299346 PMC2887065

[B62] Massad T. J. Dyer L. A. Vega C. G. (2012). Costs of defense and a test of the carbon-nutrient balance and growth-differentiation balance hypotheses for two co-occurring classes of plant defense. PLoS ONE 7:e47554. 10.1371/journal.pone.0047554 23115654 PMC3480385

[B63] Mathioni S. M. Bel ó. A. Rizzo C. J. Dean R. A. Donofrio N. M. (2011). Transcriptome profiling of the rice blast fungus during invasive plant infection and *in vitro* stresses. BMC Genomics 12:049. 10.1186/1471-2164-12-49 21247492 PMC3037901

[B64] McGrath K. C. Dombrecht B. Manners J. M. Schenk P. M. Edgar C. I. Maclean D. J.. (2005). Repressor- and activator-type ethylene response factors functioning in jasmonate signaling and disease resistance identified via a genome-wide screen of Arabidopsis transcription factor gene expression. Plant Physiol. 139, 949–959. 10.1104/pp.105.068544 16183832 PMC1256008

[B65] Morris J. H. Apeltsin L. Newman A. M. Baumbach J. Wittkop T. Su G.. (2011). clusterMaker: a multi-algorithm clustering plugin for Cytoscape. BMC Bioinformatics 12:436. 10.1186/1471-2105-12-436 22070249 PMC3262844

[B66] Nandi A. Moeder W. Kachroo P. (2005). Arabidopsis ssi2-conferred susceptibility to Botrytis cinerea is dependent on EDS5 and PAD4. Mol. Plant Pathol. 18, 363–370. 10.1094/MPMI-18-0363 15828688

[B67] von Wirén N. Lauter F. R. Ninnemann O. Gillissen B. Walch-Liu P. Engels C.. (2000). Differential regulation of three functional ammonium transporter genes by nitrogen in root hairs and by light in leaves of tomato. Plant J. 21, 167–175. 10.1046/j.1365-313x.2000.00665.x 10743657

[B68] Obayashi T. Okamura Y. Ito S. Tadaka S. Aoki Y. Shirota M.. (2014). ATTED-II in 2014: evaluation of gene coexpression in agriculturally important plants. Plant Cell Physiol. 55, e6(1–7). 10.1093/pcp/pct178 24334350 PMC3894708

[B69] Olesen J. E. Jørgensen L. N. Petersen J. Mortensen J. V. (2003). Effects of rates and timing of nitrogen fertilizer on disease control by fungicides in winter wheat. 2. Crop growth and disease development. J. Agr. Sci. 140, 15–29. 10.1017/S0021859602002897

[B70] Pageau K. Reisdorf-Cren M. Morot-Gaudry J.-F. Masclaux-Daubresse C. (2006). The two senescence-related markers, GS1 (cytosolic glutamine synthetase) and GDH (glutamate dehydrogenase), involved in nitrogen mobilization, are differentially regulated during pathogen attack and by stress hormones and reactive oxygen species in Nicotiana tabacum L. leaves. J. Exp. Bot. 57, 547–557. 10.1093/jxb/erj035 16377736

[B71] Pan X. Zhu B. Luo Y. Fu D. (2013). Unraveling the protein network of tomato fruit in response to necrotrophic phytopathogenic Rhizopus nigricans. PLoS ONE 8:e73034. 10.1371/journal.pone.0073034 24023804 PMC3759434

[B72] Pieterse C. M. J. Leon-Reyes A. Van der Ent S. Van Wees S. C. M. (2009). Networking by small-molecule hormones in plant immunity. Nat Chem Biol. 5, 308–316. 10.1038/nchembio.164 19377457

[B73] Poultney C. S. Gutiérrez R. A. Katari M. S. Gifford M. L. Paley W. B. Coruzzi G. M.. (2007). Sungear: interactive visualization and functional analysis of genomic datasets. Bioinformatics 23, 259–261. 10.1093/bioinformatics/btl496 17018536

[B74] Pusztahelyi T. Holb I. J. Pócsi I. (2015). Secondary metabolites in fungus-plant interactions. Front. Plant Sci. 6:573. 10.3389/fpls.2015.00573 26300892 PMC4527079

[B75] Reid K. E. Olsson N. Schlosser J. Peng F. (2006). An optimized grapevine RNA isolation procedure and statistical determination of reference genes for real-time RT-PCR during berry development. BMC Plant Biol. 6:27. 10.1186/1471-2229-6-27 17105665 PMC1654153

[B76] Rizhsky L. Davletova S. Liang H. Mittler R. (2004). The zinc finger protein Zat12 is required for cytosolic ascorbate peroxidase 1 expression during oxidative stress in Arabidopsis. J. Biol. Chem. 279, 11736–11743. 10.1074/jbc.M313350200 14722088

[B77] Rojas C. M. Senthil-Kumar M. Tzin V. Mysore K. S. (2014). Regulation of primary plant metabolism during plant-pathogen interactions and its contribution to plant defense. Front. Plant Sci. 5:17. 10.3389/fpls.2014.00017 24575102 PMC3919437

[B78] Scalschi L. Sanmartín M. Camañes G. Troncho P. Sánchez-Serrano J. J. García-Agustín P.. (2015). Silencing of OPR3 in tomato reveals the role of OPDA in callose deposition during the activation of defense responses against Botrytis cinerea. Plant J. 81, 304–315. 10.1111/tpj.12728 25407262

[B79] Schneider C. A. Rasband W. S. Eliceiri K. W. (2012). NIH Image to ImageJ: 25 years of image analysis. Nat. Methods 9, 671–675. 10.1038/nmeth.2089 22930834 PMC5554542

[B80] Seifi H. De Vleesschauwer D. Aziz A. Höfte M. (2014). Modulating plant primary amino acid metabolism as a necrotrophic virulence strategy: the immune-regulatory role of asparagine synthetase in Botrytis cinerea-tomato interaction. Plant Signal. Behav. 9:e27995. 10.4161/psb.27995 24521937 PMC4091234

[B81] Seifi H. S. Curvers K. De Vleesschauwer D. Delaere I. Aziz A. Höfte M. (2013). Concurrent overactivation of the cytosolic glutamine synthetase and the GABA shunt in the ABA-deficient sitiens mutant of tomato leads to resistance against Botrytis cinerea. New Phytol. 199, 490–504. 10.1111/nph.12283 23627463

[B82] Shannon P. Markiel A. Ozier O. Baliga N. S. Wang J. T. Ramage D.. (2003). Cytoscape: a software environment for integrated models of biomolecular interaction networks. Genome Res. 13, 2498–2504. 10.1101/gr.1239303 14597658 PMC403769

[B83] Smith J. E. Mengesha B. Tang H. Mengiste T. Bluhm B. H. (2014). Resistance to Botrytis cinerea in Solanum lycopersicoides involves widespread transcriptional reprogramming. BMC Genomics 15:334. 10.1186/1471-2164-15-334 24885798 PMC4035065

[B84] Snoeijers S. S. Pérez-García A. Joosten M. H. A. J. De Wit P. J. G. M. (2000). The effect of nitrogen on disease development and gene expression in bacterial and fungal plant pathogens. Eur. J. Plant Pathol. 106, 493–506. 10.1023/A:1008720704105

[B85] Su G. Kuchinsky A. Morris J. H. States D. J. Meng F. (2010). GLay: community structure analysis of biological networks. Bioinformatics 26, 3135–3137. 10.1093/bioinformatics/btq596 21123224 PMC2995124

[B86] Thimm O. Bläsing O. Gibon Y. Nagel A. Meyer S. Krüger P.. (2004). mapman: a user−driven tool to display genomics data sets onto diagrams of metabolic pathways and other biological processes. Plant J. 37, 914–939. 10.1111/j.1365-313X.2004.02016.x/pdf 14996223

[B87] Thomma B. P. Penninckx I. A. Cammue B. P. Broekaert W. F. (2001). The complexity of disease signaling in Arabidopsis. Curr Opin Immunol. 13, 63–68. 10.1016/S0952-7915(00)00183-7 11154919

[B88] Torres M. A. Jones J. D. G. Dangl J. L. (2006). Reactive oxygen species signaling in response to pathogens. Plant Physiol. 141, 373–378. 10.1104/pp.106.079467 16760490 PMC1475467

[B89] Vanacker H. Sandalio L. Jiménez A. Palma J. M. Corpas F. J. Meseguer V.. (2006). Roles for redox regulation in leaf senescence of pea plants grown on different sources of nitrogen nutrition. J. Exp. Bot. 57, 1735–1745. 10.1093/jxb/erl012 16760420

[B90] Van Baarlen P. Staats M. Van Kan J. A. (2004). Induction of programmed cell death in lily by the fungal pathogen Botrytis elliptica. Mol. Plant Pathol. 5, 559–574. 10.1111/J.1364-3703.2004.00253.X 20565630

[B91] Van der Does D. Leon-Reyes A. Koornneef A. Van Verk M. C. Rodenburg N. Pauwels L.. (2013). Salicylic acid suppresses jasmonic acid signaling downstream of SCFCOI1-JAZ by targeting GCC promoter motifs via transcription factor ORA59. Plant Cell 25, 744–761. 10.1105/tpc.112.108548 23435661 PMC3608790

[B92] Vidal E. A. Araus V. Lu C. Parry G. Green P. J. Coruzzi G. M.. (2010). Nitrate-responsive miR393/AFB3 regulatory module controls root system architecture in Arabidopsis thaliana. Proc. Natl. Acad. Sci. U.S.A. 107, 4477–4482. 10.1073/pnas.0909571107 20142497 PMC2840086

[B93] Vidal E. A. Moyano T. C. Canales J. Gutiérrez R. A. (2014). Nitrogen control of developmental phase transitions in Arabidopsis thaliana. J. Exp. Bot. 65, 5611–5618. 10.1093/jxb/eru326 25129132

[B94] Vlot A. C. Dempsey D. A. Klessig D. F. (2009). Salicylic Acid, a multifaceted hormone to combat disease. Annu. Rev. Phytopathol. 47, 177–206. 10.1146/annurev.phyto.050908.135202 19400653

[B95] Walters D. Heil M. (2007). Costs and trade-offs associated with induced resistance. Physiol. Mol. Plant P. 71, 3–17. 10.1016/j.pmpp.2007.09.008

[B96] Walters D. R. Bingham I. J. (2007). Influence of nutrition on disease development caused by fungal pathogens: implications for plant disease control. Ann. Appl. Biol. 151, 307–324. 10.1111/j.1744-7348.2007.00176.x

[B97] Wang R. Okamoto M. Xing X. Crawford N. M. (2003). Microarray analysis of the nitrate response in Arabidopsis roots and shoots reveals over 1,000 rapidly responding genes and new linkages to glucose, trehalose-6-phosphate, iron, and sulfate metabolism. Plant Physiol. 132, 556–567. 10.1104/pp.103.021253 12805587 PMC166997

[B98] Wang R. Tischner R. Gutiérrez R. A. Hoffman M. Xing X. Chen M.. (2004). Genomic analysis of the nitrate response using a nitrate reductase-null mutant of Arabidopsis. Plant Physiol. 136, 2512–2522. 10.1104/pp.104.044610 15333754 PMC523318

[B99] Wang Y. H. Garvin D. F. Kochian L. V. (2001). Nitrate-induced genes in tomato roots. Array analysis reveals novel genes that may play a role in nitrogen nutrition. Plant Physiol. 127, 345–359. 10.1104/pp.127.1.345 11553762 PMC117990

[B100] Ward J. L. Forcat S. Beckmann M. Bennett M. Miller S. J. Baker J. M.. (2010). The metabolic transition during disease following infection of Arabidopsis thaliana by Pseudomonas syringae pv. tomato. Plant J. 63, 443–457. 10.1111/j.1365-313X.2010.04254.x 20497374

[B101] Weirauch M. T. Yang A. Albu M. Cote A. G. Montenegro-Montero A. Drewe P.. (2014). Determination and inference of eukaryotic transcription factor sequence specificity. Cell 158, 1431–1443. 10.1016/j.cell.2014.08.009 25215497 PMC4163041

[B102] Windram O. Madhou P. McHattie S. Hill C. Hickman R. Cooke E.. (2012). Arabidopsis defense against Botrytis cinerea: chronology and regulation deciphered by high-resolution temporal transcriptomic analysis. Plant Cell 24, 3530–3557. 10.1105/tpc.112.102046 23023172 PMC3480286

[B103] Windram O. Penfold C. A. Denby K. J. (2014). Network modeling to understand plant immunity. Annu. Rev. Phytopathol. 52, 93–111. 10.1146/annurev-phyto-102313-050103 24821185

[B104] Zhang G.-B. Yi H.-Y. Gong J.-M. (2014). The Arabidopsis ethylene/jasmonic acid-NRT signaling module coordinates nitrate reallocation and the trade-off between growth and environmental adaptation. Plant Cell 26, 3984–3998. 10.1105/tpc.114.129296 25326291 PMC4247569

[B105] Zhang H. Rong H. Pilbeam D. (2007). Signalling mechanisms underlying the morphological responses of the root system to nitrogen in Arabidopsis thaliana. J. Exp. Bot. 58, 2329–2338. 10.1093/jxb/erm114 17578866

