# Supplementary figures and images for: Transcriptome analysis reveals regulatory networks underlying differential susceptibility to Botrytis cinerea in response to nitrogen availability in Solanum lycopersicum
Source: Front Plant Sci. 2015 Nov 4;6:911. doi: 10.3389/fpls.2015.00911 (PMC4631835; doi:10.3389/fpls.2015.00911)

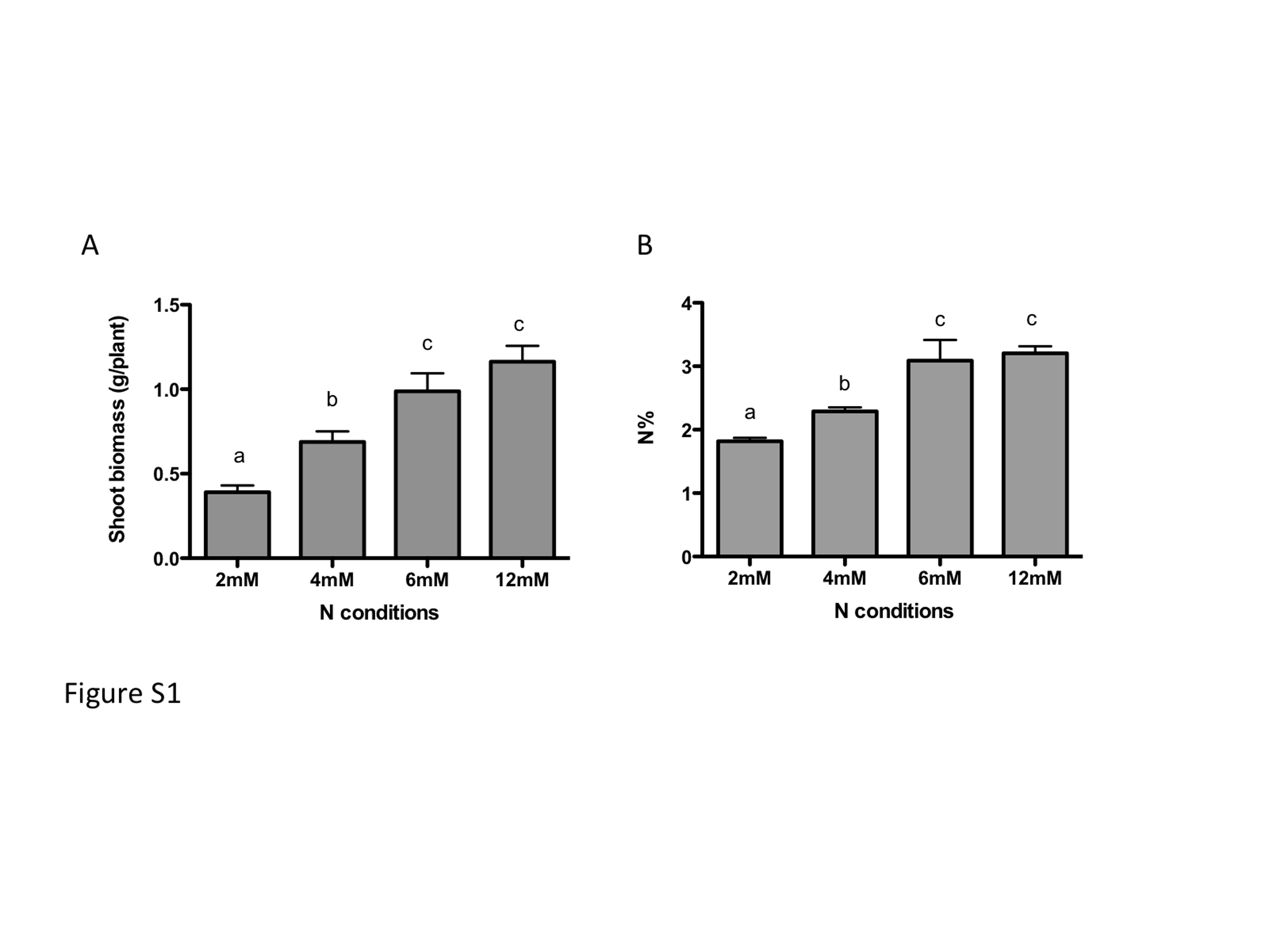

Supplement: Figure S1 — Tomato plants grown under contrasting N conditions. (A) Shoots biomass (average) of MicroTom tomato plants (4-weeks-old) grown under contrasting N conditions. (B) N percentage of plants, determined as dry weight (see Materials and Methods; n = 4). Different letters indicate significant differences among treatments (p ≤ 0.05; error bars indicate SEM). [file Image1.TIF]

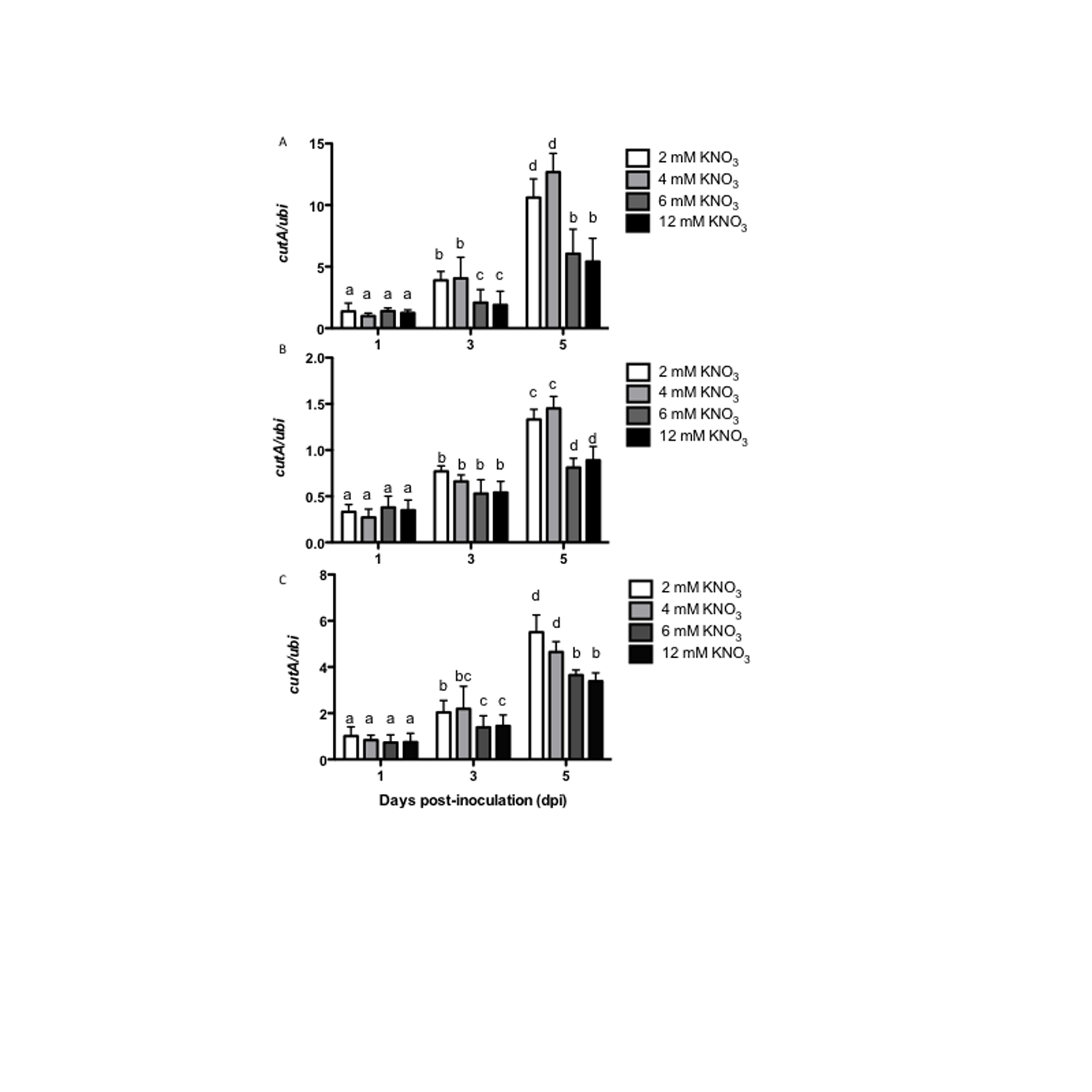

Supplement: Figure S2 — Fungal growth quantification over a B. cinerea infection time-course. The abundance of the fungus was quantified by qPCR amplification of cutA (see Materials and Methods) in infected leaves samples (A), and MG (B) and RR (C) fruits. Different letters indicate significant differences among treatments (p ≤ 0.05; error bars indicate SEM; n = 3). [file Image2.TIFF]

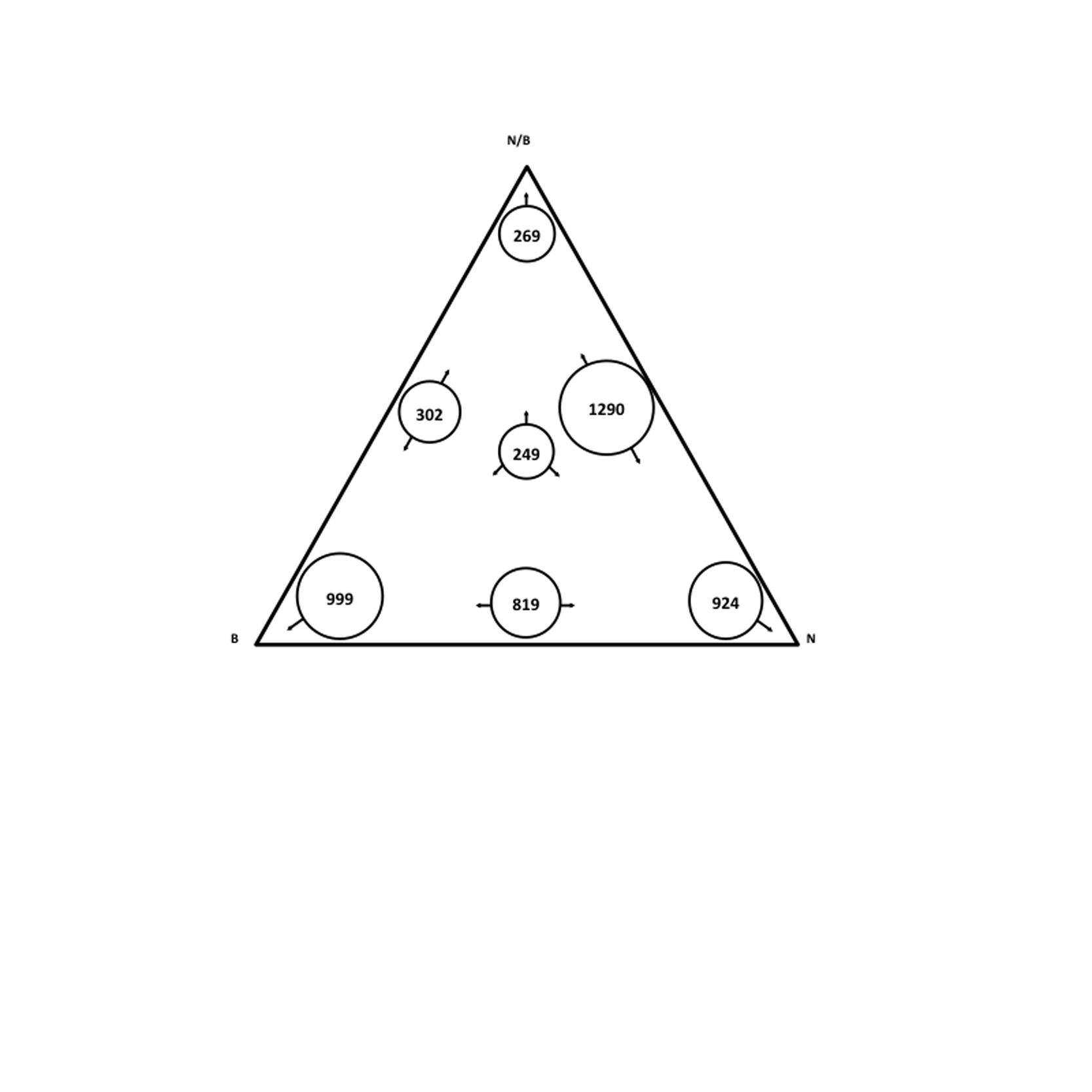

Supplement: Figure S3 — ANOVA analysis of microarray gene expression data. The number of genes with significant factors obtained by Two-way ANOVA analysis of global expression data is represented (p < 0.01), employing the SUNGEAR tool (Poultney et al., 2007). The triangle and its vertices represent the analyzed factors: B. cinerea (B), N conditions (N) and the interaction between these two factors (N/B). Circles (and their respective size) represent the number of genes controlled by the different factors, as indicated by the arrows around the circles. [file Image3.TIFF]
